# Determination of Metal Content by Inductively Coupled Plasma-Mass Spectrometry in Polish Red and White Wine Samples in Relation to Their Type, Origin, Grape Variety and Health Risk Assessment

**DOI:** 10.3390/foods12173205

**Published:** 2023-08-25

**Authors:** Dorota Jakkielska, Ioannis Dasteridis, Maciej Kubicki, Marcin Frankowski, Anetta Zioła-Frankowska

**Affiliations:** 1 Department of Analytical Chemistry, Faculty of Chemistry, Adam Mickiewicz University, Uniwersytetu Poznańskiego 8, 61-614 Poznań, Poland; dorota.jakkielska@amu.edu.pl; 2 Department of Analytical and Environmental Chemistry, Faculty of Chemistry, Adam Mickiewicz University, Uniwersytetu Poznańskiego 8, 61-614 Poznań, Poland; ziola.a@gmail.com (I.D.); marcin.frankowski@amu.edu.pl (M.F.); 3 Department of Crystallography, Faculty of Chemistry, Adam Mickiewicz University, Uniwersytetu Poznańskiego 8, 61-614 Poznań, Poland; mkubicki@amu.edu.pl

**Keywords:** multi-elemental analysis, wine, ICP-MS, ICP-OES, chemometric methods, health risk assessment

## Abstract

The main objective of the research was to assess the influence of selected factors (type of wine, grape variety, origin, alcohol content and daily consumption) on the concentration levels of 26 elements in 53 Polish wine samples, also using chemometric analysis tools. Concentration of Al, As, B, Ba, Be, Cd, Co, Cr, Cu, Fe, Hg, Li, Mn, Ni, Pb, Sb, Se, Sr, Ti, V, Zn and Zr was analyzed by ICP-MS, while concentration of Ca, Na, K and Mg was determined by ICP-OES. White wines were characterized by higher concentrations of Al, As, Be, Ca, Co, Cu, Fe, Hg, Li, Mg, Na, Pb, Sb, Ti, V, Zn and Zr (mean values: 0.075–86,403 μg·L^−1^ in white wines, 0.069–81,232 μg·L^−1^ in red wines). Red wines were characterized by higher concentrations of Ba, Cd, Cr, K, Mn, Se and Sr (mean values: 0.407–1,160,000 μg·L^−1^ in white wines, 0.448–1,521,363 μg·L^−1^ in red wines). The results obtained for the health risk assessment indices, including the Target Hazard Quotient (THQ, mean values per glass of wine: 2.097 × 10^−5^ (Cr)—0.041 (B) in all wines), indicate that the analyzed elements do not show a potential toxic effect resulting from wine consumption. The chemometric analysis confirmed that elements such as Li, Ti, Ca, Mn, Sr, Ba, Zn, Mg, Cu, Se and B were closely related to local conditions and soil properties, and the presence of Fe, Cr, V and Pb was related to contamination of the soil.

## 1. Introduction

Wine is one of the most popular alcoholic beverages in the world. According to data published by the Center for Public Opinion Research (CBOS) in 2019, the most popular alcohol beverages in Poland were beer (39% of alcohol drinkers), wine (25%) and vodka (16%) [1]. The average yearly consumption of wine and mead per person in Poland grew from 2018 to 2021 and was reported at a level of 6.7 L in 2021 [2]. MAKRO, in 2018, reported that Polish people drank mostly red wines (66%), then white wines (34%) and rose wines (21%). They preferred semi-sweet (40%) and semi-dry (39%); sweet wines were the least popular (18%). Additionally, Polish wine consumers chose mostly desert (32%) and table wines (30%); sangria (10%) and sparkling wines (9%) were not as popular. When it came to grape variety, Polish people did not pay much attention to it, and only 45% of people considered grape variety as one of their wine selection criteria, while taste, dryness, price and color were given more often—92, 86, 82 and 79%, respectively. The most popular grape varieties were Merlot (15%), Chardonnay (11%), Sauvignon Blanc (10%) and Cabernet Sauvignon (9%) [3]. Kondrat Wina Wybrane, a Polish wine brand, published a report in 2019 about the consumption of their own wines. Their consumers mostly bought red wines (52%), 95% of them being dry wines, with 4% semi-sweet and 1% sweet wines. Red wines produced from only one grape variety, such as Primitivo, Tempranillo, Monastrell, Syrah and Malbec, were the most popular choices. White wines accounted for 44% of all wines sold. Those were mostly dry (74.4% of white wines) and semi-dry (17.6%), with semi-sweet (7.8%) and sweet wines (3.16%) being less popular among Polish people. Similarly, to red wines, consumers mostly bought white wines produced just from one grape variety, such as Macabeo, Sauvignon Blanc, Viognier and Chardonnay. It is worth noting that women bought wine more often; they accounted for 58% of the buyers [4].

As reported by the National Support Center for Agriculture (KOWR), production of wine in Poland continued to grow from 2011/2012 up to 2021/2022 and was estimated at 18,444.79 hL (6358.08 hL of red wine and 12,083.71 hL of white wine) in 2021/2022. At the same time, there were 380 producers of wine in Poland, and the cultivated area of grapevines was reported to be 619.42 ha; both values have been continuously growing since 2010/2011 [5]. Poland is part of wine-growing zone A, the coldest wine-growing zone in Europe, as stated by Council Regulation (EC) No 491/2009 of 25 May 2009, amending Regulation (EC) No 1234/2007 establishing a common organization of agricultural markets and on specific provisions for certain agricultural products [6]. Vineyards can be found in all 16 voivodeships, with their highest concentration in Western Pomerania, Lubusz, Lower Silesia, Lesser Poland and Subcarpathia voivodeships, and in the Sandomierz region and Vistula River Gorge of Lesser Poland [7]. The Polish climate, being colder and more austere compared to more typical and traditional wine regions, can cause lower sugar content, usually ranging from 17 to 23%, in grapes grown in Poland, which leads to higher acidity and lower alcohol content in the wine produced. Such wine, especially white wine, is fresh and crisp in taste [8]. Adding to that, the Polish climate is also very diverse, with access to the sea in the north and vast mountains in the south, which leads to many regions in Poland having their own unique microclimate, called “terroir”. Polish vineyards are mainly family businesses, and their own unique style of wine is a result of years spent searching for a suitable place to grow grapes and depends on the grape varieties and types of wines produced. All of that can lead to differences not only between wines produced in Poland and other countries but also between wines produced in different regions of Poland.

Wine contains not only water, sugar and alcohol but also a great variety of organic and inorganic components, such as amino acids, vitamins, polyphenols and minerals [9,10]. It is believed that moderate wine consumption could have positive effects on human health and contribute to the daily intake of essential elements [9,11]. However, wine can also be a source of potentially toxic metals and metalloids, such as As, Cd and Pb [9]. The metal composition of wine affects its organoleptic characteristics, such as aroma, color and mouth feel, and its qualitative characteristics, such as alcohol and total acidity. The sources of metals in wine can be natural, such as the absorption of metals from vineyard soil, the location of the vineyard, and the absorption properties of used grape varieties, or anthropogenic, due to contamination and pollution, such as environmental pollution, agrochemical treatments and absorption from used equipment. The change in the metal profile can be a consequence of filtering, pH adjustment, yeast hull addition, fining, fermentation storage conditions and packaging materials. The elemental content can and has been successfully used to distinguish the geographic origin and country of wine and to discriminate intraregional wines in multiple studies [11,12,13].

The popularity of wine around the world and the potential presence of toxic metals show the importance of monitoring the potential risk to human health from wine consumption. This study aimed to assess the health risk of consuming Polish wine and to analyze the metal content of Polish wine and its connection with used grape varieties, other fruits, and the region of growing and production. Moreover, this work aimed to apply chemometric techniques to find specific relationships between the wine samples and/or between the chemical variables in order to classify the wines according to their metal content. That approach would allow us to show the discriminating chemical indicators for each group of clustered wine types. It is worth underlining that the major goals of using multivariate data are the identification of groups of similarity (clusters) between the objects of the study (in this study, wine samples) or between the descriptor variables. To interpret the analytical data from the multielement analysis of wine samples, the chemometric methods principal components analysis (PCA) and cluster analysis (hierarchical cluster analysis—HCA) were used. Both methods have been used many times in determining the profiles of the content of organic and inorganic compounds in various types of wines [14,15,16,17,18,19], and they are both well-known and documented and do not need detailed descriptions [17,20].

## 2. Materials and Methods

### 2.1. Wine Samples

Fifty-three bottles of wine, from various grape varieties and fruits, produced in eight voivodeships of Poland: Lesser Poland, Lower Silesian, Lublin, Lubusz, Silesian, Subcarpathian, Świętokrzyskie (Holy Cross) and West Pomeranian, were obtained for this study. It was important to obtain wines from different regions because differences in type and quality of soil and height above the sea impact the elemental content of grapes, other fruits and the product that is wine. There were 31 white wines and 22 red wines. Most of the wines, 31, were produced from one of the following grape varieties: Cabernet Cortis, Chardonnay, Gewürztraminer, Hibernal, Pinot Gris, Pinot Noir, Regent, Riesling, Rondo, Seyval Blanc, Sibera, Solaris and Zweigelt. Eighteen wines were produced from more than one grape variety, and 4 wines were produced from the following fruits: cherry, pear, apple and black currant. The characterization of Polish wine samples is given in Table S1 of the Supplementary Materials.

### 2.2. Reagents and Standards

For the calibration step of ICP-MS, various standards were used, including the ICP IV multi-element standard (1000 mg·L^−1^; each element) (Merck, New York, NY, USA) and single standards (1000 mg·L^−1^; each element): As, Sb, Se, Mo and V (Sigma-Aldrich, New York, NY, USA) and Hg (Merck, USA). Additionally, as internal standards, Sc, Rh, Tb and Ge in supra-pure 1% HNO3 (Merck, New York, NY, USA) and deionized water collected from the Milli-Q Direct 8 Water Purification System (Merck Millipore, New York, NY, USA) were used for all solution preparation. For the calibration of ICP-OES, the single standards (1000 mg·L^−1^; each element) of Ca, Na, Mg and K were used (Sigma-Aldrich, New York, NY, USA).

### 2.3. Wine Analysis

Concentrations of Al, As, B, Ba, Be, Cd, Co, Cr, Cu, Fe, Hg, Li, Mn, Ni, Pb, Sb, Se, Sr, Ti, V, Zn and Zr were analyzed by the ICP-MS analytical technique (ICP-MS 2030, Shimadzu, Kioto, Japan). While concentrations of Ca, Na, K and Mg were obtained by the ICP-OES (Shimadzu ICPE-9820, Kioto, Japan) analytical technique with the application of a previously published procedure [21]. Wine samples were diluted 10 times with deionized water without any prior preparation. Calibration curves for each element were obtained using 9 different concentration levels. For ICP-MS standards containing 0.01, 0.25, 0.1, 0.25, 1.00, 2.5, 10.0, 25.0 and 100 µg·L^−1^ and for ICP-OES standards containing 1.0, 2.5, 10.0, 25.0, 100, 250, 1000 µg·L^−1^ and 2.5 and 10.0 mg·L^−1^. Depending on the signal from the ICP, the range of the standard curve was adjusted for both techniques. For ICP-MS in the range of low signals of analyzed elements. During the calibration step, the accuracy of the analyses was checked by means of certified reference materials: trace metals ICP—sample 1 and trace metals ICP—sample 2. The recovery obtained for selected elements in both CRMs ranged from 96% to 104%. In addition, in order to check the influence of the matrix, an analysis of standard solutions prepared by dilution with deionized water and alcohol (1.2% C_2_H_5_OH) was performed. No significant differences were observed in the obtained analytical signals for both types of standards.

Validation of the ICP-MS analytical technique was performed using the ICP-MS tuning solution from Roth (Roth, Germany). Validation was carried out for the masses Be_9_, In_115_ and Bi_209_ in terms of minimum signal reciprocity, RSD, resolution, background measurement and the content of double-charged ions and oxides for conditions without collision gas and for Co_59_ in the conditions of collision cell operation, background measurement in these conditions, as well as the content of double-charged ions and oxides. During the analysis of the wine samples, all parameters were successfully obtained, and the entire validation test was successfully completed. The limits of detection (LOD) and limit of quantification (LOQ) were calculated as three times signal to noise (LOD) and ten times signal to noise (LOQ). The operating conditions for the wine analysis for both ICP spectrometers are presented in Table 1, and the analytical performances of the methods applied for multielement analysis are given in Table S2 of the Supplementary Materials.

### 2.4. Chemometric Analysis

The principal component analysis (PCA) of the standardized measurements was applied to establish a correlation between wine samples according to their elemental composition, origin of these wines, type of wine (red or white), and grape varieties. In addition, Hierarchical Cluster Analysis was performed after z-transformation of the input raw data (Ward’s method of linkage) and cluster significance by Sneath’s index. In the case of missing data (under the limit of detection), the value of LOD/2 was introduced. Data analysis have been carried out by means of the STATISTICA 8.0 (New York, NY, USA) software package.

### 2.5. Health Risk Assessment

The average daily intake of elements (*ADI*) for adults was estimated using Equation (1):

*ADI* = *Cm* × *IRn* × *EFh* × *EDf*/*Baw* × *ATd*, (1)

where *Cm* is the element concentration in wine samples (µg·L^−1^); *IRn* is the daily intake of wine in L/person/day; *EFh* is the exposure frequency (365 days/year); *EDf* is the exposure duration (70 years); *Baw* is the average body weight (in this study, 70 kg was considered average), and *ATd* is the carcinogenic effect based on average exposure time (*EFh* × *EDf*) [22].

In evaluating the non-carcinogenic health risks due to the consumption of wine, the target hazard quotient (*THQ*) was calculated based on Equation (2):

*THQ* = *ADI*/*RfD_ing_*, (2)

where *RfD_ing_* is the oral/ingestion reference dose (µg·kg^−1^·day^−1^), which is used for non-cancer health assessment [22].

The carcinogenic risk (*CR*) of elements caused by the probability of getting sick from cancer due to the direct ingestion of wine was calculated by Equation (3):

*CR* = *ADI* × *CSF_ing_*, (3)

where *CSF_ing_* is the ingestion cancer slope factor (mg·kg^−1^·day^−1^)^−1^ [22].

To estimate the cumulative potential health risk caused by a mixture of elements due to the consumption of wine, the hazard index (*HI*) based on the sum of the particular *THQ* of each element was applied as indicated in Equation (4):

*HI* = *THQ*_1_ + *THQ*_2_ + *THQ*_3_ + … + *THQ_n_*. (4)

## 3. Results and Discussion

**Figure 1:**
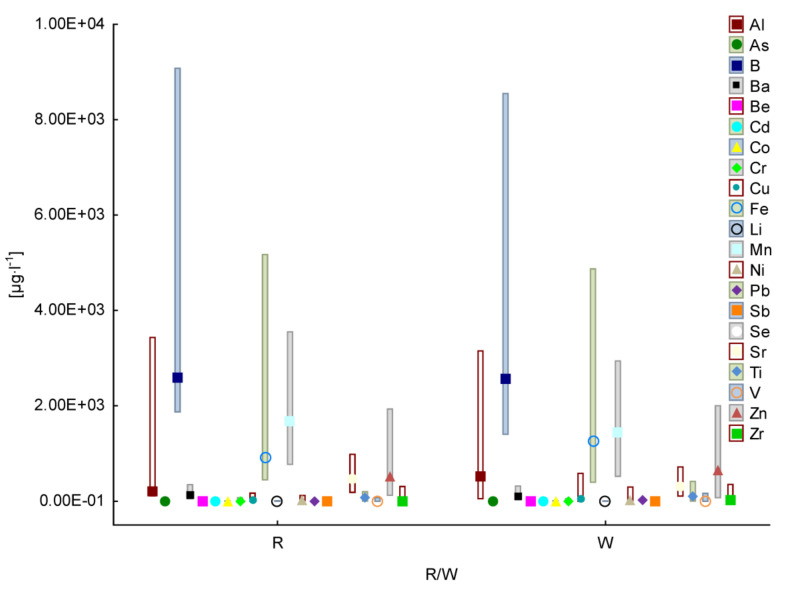
Average and min–max values for Al, As, B, Ba, Be, Cd, Co, Cr, Cu, Fe, Li, Mn, Ni, Pb, Sb, Se, Sr, Ti, V, Zn and Zr concentrations in red (R) and white (W) Polish wines.

In this study, the concentration of 26 elements—Al, As, B, Ba, Be, Ca, Cd, Co, Cr, Cu, Fe, Hg, K, Li, Mg, Mn, Na, Ni, Pb, Sb, Se, Sr, Ti, V, Zn and Zr—was determined in 53 Polish wines—22 red wines and 31 white wines. Figure 1 presents the results without the values for the macroelements due to the much higher concentrations, which are presented in Table S3 of the Supplementary Materials.

The highest total concentrations of all elements were obtained for samples of Ice Wine (no. 3-B, 3.672 g·L^−1^) and Pinot Noir 2013 (no. 26A, 2.520 g·L^−1^). The lowest total concentrations of all elements were obtained for samples of Chardonnay (no. 28B, 0.335 g·L^−1^) and Solaris 2014 (no. 31B, 0.750 g·L^−1^). Not all metals were determined in all samples. The mean metal concentrations for all wine samples can be arranged in the following order: Hg < 0.1 μg·L^−1^ < Se < Sb < Cd < 1 μg·L^−1^ < Be < Li < Co < As < 10 μg·L^−1^ < Pb < Cr < V < Ni < Zr < Cu < 100 μg·L^−1^ < Ti < Ba < Sr < Zn < Al < 1000 μg·L^−1^ < Fe < Mn < B < 10 mg·L^−1^ < Na < Ca < Mg < 100 mg·L^−1^ < K.

Analyzed metals can be divided into four groups according to their required consumption in the human diet and based on deficiency and toxicity concentrations: macrominerals, essential minerals, trace metals and toxic metals [23].

Out of all the analyzed metals, four can be considered macrominerals: Ca, K, Mg and Na. Total concentrations of macrominerals ranged from 0.326 g·L^−1^ (no. 28B) to 3.652 g·L^−1^ (no. 3-B). The average total concentration of macrominerals in all samples was 1.478 g·L^−1^. The lowest mean concentration was obtained for Na (37.20 mg·L^−1^) and the highest for K (1.310 g·L^−1^). Mean macromineral concentrations can be arranged in order: Na < Ca < Mg < 100 mg·L^−1^ < K. According to the significance of macrominerals for wine quality parameters, we observed in this study the highest content of potassium in both types of wine. It should be underlined that potassium is the most common cause of instability, which is related to the natural constituent of grapes and its presence in wine. High concentrations of potassium in wine can lead to an increase in its acidity, which is associated with the formation of tartaric-potassium salts and inhibition of the degradation of malic acid [14]. However, the presence of potassium in wine can be associated with the variety of grapes, soil, climatic conditions, time of harvest, temperature of fermentation, type of storage and conditions of pH reaction [24]. Additionally, it was found that a high content of calcium may result in the formation of crystalline calcium L-tartrate. Calcium concentrations above 80 mg·L^−1^ in white wines and 60 mg·L^−1^ in red wines are considered to be at risk of sediment formation [14]. In our study, we obtained the content of Ca above 60 mg·L^−1^ for 14 red wine samples, with a median value of 66.70 mg·L^−1^. In the case of white wine samples, Ca content above 80 mg·L^−1^ was found in 15 samples, with a median value of 73.90 mg·L^−1^. Calcium concentrations determined in both types of wine may indicate a high probability of instability, which may lead to the formation of sediment in the analyzed wines after a longer period of bottle aging.

Total concentrations of essential minerals—B, Co, Cr, Cu, Fe, Li, Mn, Se, V and Zn— ranged from 3.796 mg·L^−1^ (no. 3B) to 15.20 mg·L^−1^ (no. 3B), and the average total concentration of essential minerals was 7.815 mg·L^−1^. Mean essential mineral concentrations can be arranged in order: Se < 1 μg·L^−1^ < Li < Co < 10 μg·L^−1^ < Cr < V < Cu < 100 μg·L^−1^ < Zn < 1000 μg·L^−1^ < Fe < Mn < B. According to the highest content of B, it should be underlined that it is one of the essential nutrients for plants, like potassium. The contribution of boron can be linked to the types of fertilization practices and plant uptake processes [24]. In the case of copper, which can also contribute to the instability of the wine, which is characterized by haze that turns into sediment, a value of 0.5 mg·L^−1^ is considered safe [14]. Moreover, the source of copper in wine is linked with its use as a plant protection substance and fungicide, a mixture of copper sulfate with calcium hydroxide called the Bordeaux mixture, which is not allowed to be used in the European Community [24]. Additionally, copper can be added to wine to remove organic sulfur odors from the fermentation and bottling processes. That is why a Cu content greater than 1 mg·L^−1^ can cause a metallic bitter taste and turbidity in wine [25]. Copper above 0.5 mg·L^−1^ was not determined in the analyzed red wines. On the other hand, for white wines, the concentration of copper above the safe limit was determined for two samples, and for the remaining ones, the content was below 0.5 mg·L^−1^ (Table S3). This may indicate a low probability of copper-induced instability in the analyzed wine samples. It is well known that iron, like copper and zinc, is essential for humans [26]. The occurrence of iron in wine can be associated with the type of soil and the stabilization treatments [24]. In addition, iron instability occurs when it is present at concentrations above 5 to 6 mg·L^−1^. When present at concentrations of 10 mg·L^−1^ or higher, iron can also contribute to the metallic taste of wine [14]. The analyzed wines contained iron at an average level of 1.784 mg·L^−1^ (white) and 1.329 mg·L^−1^ (red), which may indicate a low probability of danger from iron. Only sweet red wine had an iron level of 5.17 mg·L^−1^, making it potentially susceptible to iron instability. In the case of zinc, the trace concentration in wine can occur from the use of pesticides and fertilizers, like superphosphate. Additionally, the high content of Zn can affect wine quality [24]. In our study, no sample exceeded the OIV limit of 5 mg Zn·L^−1^, and the average concentration of Zn was 636 µg·L^−1^ (all 53 samples).

Out of all the analyzed metals, six can be considered trace elements: As, Cr, Ni, Sr, Ti and Zr. Their total concentrations ranged from 0.161 mg·L^−1^ (no. 20-B) to 1.562 mg·L^−1^ (no. 9B), and the average total concentration was 0.629 mg·L^−1^. The order of trace metals, based on their mean concentration, is As < 10 μg·L^−1^ < Cr < Ni < Zr < 100 μg·L^−1^ < Ti < Sr. It is important to stress that Ti can be applied as a fingerprint of soil contribution and can also be used as an additive for bottle coloring [24].

The total concentration of 10 analyzed metals that can be considered toxic is: Al, As, Ba, Be, Cd, Cr, Hg, Ni, Pb and Sb [27,28] ranged from 0.187 mg·L^−1^ (no. 4B) to 3.816 mg·L^−1^ (no. 6-A), and the average total concentration was 0.874 mg·L^−1^. The lowest mean concentration was obtained for Hg (0.073 μg·L^−1^) and the highest for Al (651.6 μg·L^−1^), and they can be arranged, based on their mean concentrations, in order: Hg < Sb < Cd < 1 μg·L^−1^ < Be < As < 10 μg·L^−1^ < Pb < Cr < Ni < 100 μg·L^−1^ < Ba < Al. It is worth emphasizing that Al, Pb and Cr can also cause instability in wine, which can cause browning, turbidity and astringency, which affect the quality and organoleptic properties of the wine [14]. In the case of aluminum, its presence can be linked to the addition of bentonite during wine processing. The presence of chromium in wine may be related to impurities resulting from the method of fermentation and the use of chromium oxides after bottling. As for lead, the main sources of its presence in wine are related to environmental pollution and the corrosion of metal wine-making equipment [25]. In the case of our research, the determined concentrations of Al, Pb and Cr were below the permissible concentration limits (8.0, 0.15 and 0.1 mg·L^−1^, respectively) [14,29], so they should not pose a threat related to the instability of the wine.

Obhodaš et al. (2021) analyzed element content in white and red wines from Austria and Croatia. Mean concentrations of K (917.2 mg·L^−1^) and Mn (0.985 mg·L^−1^) were lower in Austrian and Croatian white and red wines than in Polish white and red wines; mean concentrations of Fe (1.365 mg·L^−1^) and Cu (0.125 mg·L^−1^) were higher; and mean concentrations of Ca (66.05 mg·L^−1^), Zn (0.6465mg·L^−1^) and Sr (0.331 mg·L^−1^) were comparable [30].

Leder et al. (2021) conducted a multielement composition analysis of white and red wines from continental and coastal Croatia. Mean concentrations of As (7.5 μg·L^−1^), Ca (85.0 mg·L^−1^), Co (5.9 μg·L^−1^), Cr (19 μg·L^−1^), Cu (0.18 mg·L^−1^), Fe (1.91 mg·L^−1^), Li (4.6 μg·L^−1^), Pb (30.2 μg·L^−1^) and V (83.6 μg·L^−1^) were higher in Croatian wines than in Polish wines; mean concentrations of B (2.98 mg·L^−1^), K (788 mg·L^−1^), Mn (0.96 mg·L^−1^) and Na (14.3 mg·L^−1^) were lower in Croatian wines than in Polish wines; and concentrations of Al (0.59mg·L^−1^), Ba (0.11 mg·L^−1^), Cd (0.7 μg·L^−1^), Mg (81.3 mg·L^−1^), Sr (0.46 mg·L^−1^) and Zn (0.69 mg·L^−1^) were comparable [31].

Deng et al. (2019) analyzed trace element content in Chinese wines from different regions. Mean concentrations of Cr, Mn, As and Se were higher in Chinese wines than in Polish wines; mean concentrations of Co, Cu and Al were slightly higher in Chinese wines than in Polish wines; and concentrations of Ni, Zn, Cd and Pb were comparable [11].

### 3.1. Type of Wine—White and Red

In this study, 31 white wines and 22 red wines were analyzed, produced from various varieties of grapes and other fruits (Table S1). The highest total concentrations of all elements in white wines were obtained for Ice Wine (no. 3-B, made from apples, 3.672 g·L^−1^) and Parus A (no. 17B, 1.960 g·L^−1^). The lowest total concentrations of all elements in white wines were obtained for Chardonnay (no. 28B, 0.335 g·L^−1^) and Solaris 2014 (no. 31B, 0.750 g·L^−1^). The mean metal concentration in white wines ranged from 0.075 μg·L^−1^ (Hg) to 1.160 g·L^−1^ (K) and can be arranged in order Hg < Se < Cd < Sb < 1 μg·L^−1^ < Be < Li < Co < As < 10 μg·L^−1^ < Cr < Pb < V < Ni < Zr < 100 μg·L^−1^ < Cu < Ti < Ba < Sr < Zn < Al < 1 mg·L^−1^ < Mn < Fe < B < 10 mg·L^−1^ < Na < Ca < Mg < 1 g·L^−1^ < K. For red wines, the highest total concentration of all elements was obtained for Pinot Noir 2013 (no. 26A, 2.520 g·L^−1^) and the lowest for Geltrus XIV—2015 (12A, 1.143 g·L^−1^). The mean metal concentration in red wines ranged from 0.069 μg·L^−1^ (Hg) to 1.521 g·L^−1^ (K) and can be arranged in the following order: Hg < Be < Se < Sb < Cd < 1 μg·L^−1^ < Li < As < Co < Pb < 10 μg·L^−1^ < V < Cr < Zr < Ni < Cu < Ti < 100 μg·L^−1^ < Ba < Al < Sr < Zn < 1 mg·L^−1^ < Fe < Mn < B < 10 mg·L^−1^ < Na < Ca < Mg < 1 g·L^−1^ < K. In both white and red wines, the concentration of Hg was the lowest and the concentration of K was the highest. The mean concentrations of Al, As, Be, Ca, Co, Cu, Fe, Hg, Li, Mg, Na, Ni, Pb, Sb, Ti, V, Zn and Zr were lower in red wines than in white wines. The mean concentrations of Ba, Cd, Cr, K, Mn, Se and Sr were higher in red wines than in white wines. The mean concentrations of B were similar in red and white wines. The mean total concentrations of macrominerals and trace metals were lower in white wines than in red wines, and the mean total concentrations of essential minerals, toxic metals and heavy metals were higher in white wines compared to red wines.

In comparison, research by Gonzalez et al. (2021) showed that in Spanish wines, they did not observe any correlations between Ni and Cr contents in red and white wines, but in the case of red wines, they analyzed higher contents of Al, As, Fe, Sb and Zn. White Spanish wine samples presented higher Cd contents than red, which was linked by authors with different winemaking processes and with additives used for clarifying and stabilizing white wines. Similarly to Polish wines, in Spanish wines, higher concentrations of lead were determined in white wines than in red wines, which the authors explained by a different way of producing white wines [25].

Debastiani et al. (2021) analyzed elemental concentrations in Brazilian sparkling white wines. Mean concentrations of Na (13.9 mg·L^−1^), Mg (52.0 mg·L^−1^), K (530.7 mg·L^−1^), Ca (40.4 mg·L^−1^) and Fe (0.67 mg·L^−1^) were lower in Brazilian sparking white wines than in Polish white wines, and mean concentrations of Mn (1.63 mg·L^−1^) and Zn (0.77 mg·L^−1^) were comparable. Just like in our study, the highest mean concentration was obtained for K, but it was lower than its value in Polish wines [32].

Fermo et al. (2021) analyzed the elemental contents of Italian white, rose and red wines. In Italian white wines, mean concentrations of Sb (31.21 μg·L^−1^), Cd (0.87 μg·L^−1^), Co (3.36 μg·L^−1^), Pb (21.66 μg·L^−1^), Se (1.38 μg·L^−1^), Cu (75.84 μg·L^−1^) and Zn (829.31 μg·L^−1^) were higher than in Polish white wines, mean concentrations of As (2.38 μg·L^−1^), Ba (50.12 μg·L^−1^), Ni (16.73 μg·L^−1^), V (1.71 μg·L^−1^), Mg (62.65 mg·L^−1^), Na (18.14 mg·L^−1^), Cr (6.54 μg·L^−1^), Al (454.00 μg·L^−1^), Mn (928.68 μg·L^−1^), Fe (0.49 mg·L^−1^) and K (721.62 mg·L^−1^) were lower than in Polish white wines and mean concentration of Ca (78.11 mg·L^−1^) was similar. In Italian red wines, mean concentrations of Pb (64.06 μg·L^−1^), Sb (28.56 μg·L^−1^), Se (9.65μg·L^−1^), Na (16.88 mg·L^−1^), Co (2.57 μg·L^−1^), Zn (721.77 μg·L^−1^) and Mg (92.79 mg·L^−1^) were higher than in Polish red wines, mean concentrations of As (0.20 μg·L^−1^), Cd (0.39 μg·L^−1^), Cr (3.28 μg·L^−1^), Ni (18.00 μg·L^−1^), Al (139.38 μg·L^−1^), V (1.77 μg·L^−1^), Fe (0.85 mg·L^−1^), K (1065.07 mg·L^−1^), Cu (16.81 μg·L^−1^) and Mn (1257.25 μg·L^−1^) were lower than in Polish red wines and mean concentrations of Ba (148.38 μg·L^−1^) and Ca (63.12 mg·L^−1^) were comparable. Both in white and red Italian wines, the highest mean concentration was obtained for K, as in our study; however, the values were lower than in Polish wines [12].

In a previous study conducted for Polish white and red wines by Płotka-Wasylka et al. (2018), 44 wine bottles were analyzed. In white wines, average concentrations of As (1.4 μg·L^−1^), B (3730 mg·L^−1^), Ba (140 μg·L^−1^), Ca (7461 mg·L^−1^), Cd (1 μg·L^−1^), Cu (290 μg·L^−1^), Hg (0.4 μg·L^−1^), K (91.145 g·L^−1^), Li (5.6 μg·L^−1^), Mg (9376 mg·L^−1^), Mn (192 mg·L^−1^), Ni (64 μg·L^−1^), Pb (20 μg·L^−1^), Sb (2.3 μg·L^−1^), Se (24 μg·L^−1^) and Zn (73.4 mg·L^−1^) were higher than in our study, average concentrations of Al (710 μg·L^−1^), Co (2 μg·L^−1^), Fe (520 μg·L^−1^), Na (890 μg·L^−1^), Sr (290 μg·L^−1^), Ti (33 μg·L^−1^), V (7.5 μg·L^−1^) and Zr (5.9 μg·L^−1^) were lower than in our study, and average concentrations of Cr (14 μg·L^−1^) were similar in both studies. In red wines, average concentrations of Al (580 μg·L^−1^), As (6 μg·L^−1^), B (6870 mg·L^−1^), Ba (310 μg·L^−1^), Ca (7504 mg·L^−1^), Cu (130 μg·L^−1^), Hg (0.3 μg·L^−1^), K (182.845 g·L^−1^), Li (11.2 μg·L^−1^), Mg (10.600 g·L^−1^), Mn (318 mg·L^−1^), Ni (53 μg·L^−1^), Pb (28 μg·L^−1^), Se (3.9 μg·L^−1^) and Zn (34.8 mg·L^−1^) were higher than in this study, average concentrations of Cd (0.6 μg·L^−1^), Co (2 μg·L^−1^), Cr (12 μg·L^−1^), Fe (790 μg·L^−1^), Na (1050 μg·L^−1^), Ti (30 μg·L^−1^), V (8.2 μg·L^−1^) and Zr (2.5 μg·L^−1^) were lower than in our study, and average concentrations of Sb (0.4 μg·L^−1^) and Sr (490 μg·L^−1^) were similar in both studies. It was different for Ca, Fe, Li, Mg, Na, Pb, V, Cd, Cr and Se compared to our study [29].

Wu et al. (2021) analyzed the elemental content of French red wines available in China. Average concentrations of Mg (90.552 mg·L^−1^), B (5.179 mg·L^−1^), Al (0.738 mg·L^−1^), Cr (0.039 mg·L^−1^), Fe (2.322 mg·L^−1^) and Cu (0.109 mg·L^−1^) were higher than in Polish red wines, average concentrations of K (1089.084 mg·L^−1^), Na (18.690 mg·L^−1^), Mn (1.013 mg·L^−1^), Zn (0.441 mg·L^−1^), Sr (0.388 mg·L^−1^) and Ba (0.064 mg·L^−1^) were lower than in Polish red wines; and average concentrations of Ca (53.781 mg·L^−1^) and Ti (0.079 mg·L^−1^) were similar in Polish and French red wines [33].

Skendi et al. (2020) analyzed the metal content of Greek white and red wines. The mean concentrations of Cd (0.004, 0.002 mg·L^−1^), Pb (0.036, 0.025 mg·L^−1^) and Ni (0.157, 0.156 mg·L^−1^) were higher in Greek red and white wines than in Polish red and white wines. Mean concentrations of Cr (0.007, 0.003 mg·L^−1^), Fe (0.804, 1.103 mg·L^−1^), Mn (1.474, 0.503 mg·L^−1^), K (79.6, 70.1 mg·L^−1^), Na (2.7, 2.9 mg·L^−1^), Ca (6.1, 6.3 mg·L^−1^) and Mg (9.9, 8.9 mg·L^−1^) were lower in Greek red and white wines. It is worth noting that in Polish red and white wines, the mean concentrations of K were 1521 mg·L^−1^ and 1160 mg·L^−1^, respectively, while in Greek wines they were below 80 mg·L^−1^. The mean concentration of Cu (0.076 mg·L^−1^) was lower in Greek white wines, and the mean concentration of Zn (0.900 mg·L^−1^) was higher in Greek white wines. The mean concentrations of Cu (0.050 mg·L^−1^) and Zn (0.612 mg·mg·L^−1^) were similar in Greek and Polish red wines [34].

Fabjanowicz et al. (2022) determined concentrations of Ag, Al, As, B, Ba, Be, Ca, Cd, Co, Cr, Cu, Fe, Hg, K, Li, Mg, Mn, Na, Ni, Pb, Sb, Se, Sn, Sr, Ti, Tl, V, Zn and Zr in 10 white, 10 red and three rose wines from different regions of Poland. All elements, except Ag, Sn and Tl, were also analyzed in our study. In red wines, mean concentrations of Ca (56.27 mg·L^−1^), Co (1.966 μg·L^−1^), Hg (0.079 μg·L^−1^) and Mg (88.77 mg·L^−1^) were similar to results obtained in our study, mean concentrations of Al (301.2 μg·L^−1^), Ba (80.89 μg·L^−1^), Cd (0.247 μg·L^−1^), Cr (7.835 μg·L^−1^), Fe (885.0 μg·L^−1^), Mn (1012 μg·L^−1^), Na (5866 μg·L^−1^), Pb (3.699 μg·L^−1^), Sr (355.0 μg·L^−1^), Ti (21.21 μg·L^−1^), V (4.915 μg·L^−1^) and Zr (4.325 μg·L^−1^) were lower than values determined in our study and mean concentrations of As (17.47 μg·L^−1^), B (5122 μg·L^−1^), Be (0.322 μg·L^−1^), Cu (66.63 μg·L^−1^), K (6162 mg·L^−1^), Li (3.938 μg·L^−1^), Ni (62.75 μg·L^−1^), Sb (0.603 μg·L^−1^), Se (7.146 μg·L^−1^) and Zn (891.7 μg·L^−1^) were higher than in our study. In white wines, mean concentrations of Al (676.6 μg·L^−1^), Ca (77.45 mg·L^−1^), Co (3.189 μg·L^−1^), Cr (11.27 μg·L^−1^), Fe (1559 μg·L^−1^), Mg (83.14 mg·L^−1^), Ni (54.18 μg·L^−1^) and Sb (0.600 μg·L^−1^) were comparable to results obtained in our study, mean concentrations of Ba (47.00 μg·L^−1^), Cd (0.339 μg·L^−1^), Hg (0.034 μg·L^−1^), Mn (1060 μg·L^−1^), Na (12.68 mg·L^−1^), Pb (5.006 μg·L^−1^), Sr (247.6 μg·L^−1^), Ti (23.55 μg·L^−1^), V (12.59 μg·L^−1^) and Zr (8.638 μg·L^−1^) were lower than values determined in our study and mean concentrations of As (5.789 μg·L^−1^), B (4899 μg·L^−1^), Be (2.867 μg·L^−1^), Cu (428.1 μg·L^−1^), K (6451 mg·L^−1^), Li (4.870 μg·L^−1^), Se (2.583 μg·L^−1^) and Zn (918.6 μg·L^−1^) were higher than in our study. In both studies, mean concentrations of Al, Be, Ca, Co, Cu, Fe, Li, Na, Pb, Ti, V, Zn and Zr were higher in white wines than values determined in red wines, and mean concentrations of Ba, Se and Sr were higher in red wines than in white wines [15].

### 3.2. Grape Varieties and Other Fruits

The analyzed wines in this study were produced from 13 grape varieties: Pinot Noir (red wine), Seyval Blanc (white), Regent (red), Sibera (white), Hibernal (white), Rondo (red), Zweigelt (red), Riesling (white), Gewürztraminer (white), Cabernet Cortis (red), Chardonnay (white), Solaris (white) and Pinot Gris (white) and from four fruits—cherries, pears, apples and blackcurrant (ribes nigrum). Part of the analyzed wines were produced from at least two different varieties of grapes.

The highest total of mean concentrations of all elements were determined for fruit wines (2.207 g·L^−1^) and Pinot Noir (1.834 g·L^−1^) and the lowest for Solaris (0.750 g·L^−1^) and Chardonnay (0.890 g·L^−1^). Based on the total mean concentrations of all elements in wines, grape varieties, and fruits, they can be arranged in order: Solaris < Chardonnay < Pinot Gris < 1 g·L^−1^ < Zweigelt < Gewürztraminer < Seyval Blanc < Riesling < mixed grape varieties < Hibernal < Cabernet Cortis < 1.5 g·L^−1^ < Sibera < Rondo < Regent < Pinot Noir < 2 g·L^−1^ < fruits.

The highest mean concentration of all macrominerals was found in wines produced from fruits (2.195 g·L^−1^) and the lowest in Solaris wines (743.6 mg·L^−1^). All grape varieties and fruits can be arranged, based on mean total macromineral concentrations, in order: Solaris < Chardonnay < Pinot Gris < 1 g·L^−1^ < Zweigelt < Gewürztraminer < Seyval Blanc < Riesling < Hibernal < mixed grape varieties < Cabernet Cortis < Sibera < Rondo < Regent < Pinot Noir < 2 g·L^−1^ < fruits.

The highest mean concentration of all essential minerals was found in Pinot Gris wines (10.54 mg·L^−1^), which contained one of the lowest mean concentrations of all macrominerals, and the lowest mean total essential mineral concentration was found in Rondo wines (5.153 mg·L^−1^), which contained a high mean concentration of all macrominerals. Based on the mean concentration of all essential minerals, grape varieties and fruits can be arranged in order: Rondo < Sibera < Solaris < Zweigelt < Seyval Blanc < Regent < 6000 μg·L^−1^ < Cabernet Cortis < 7000 μg·L^−1^ < Chardonnay < Riesling < Hibernal < 8000 μg·L^−1^ < Gewürztraminer < Pinot Noir < mixed < 9000 μg·L^−1^ < fruits < Pinot Gris.

The mean concentrations of all trace elements ranged from 331.0 μg·L^−1^ to 1562 μg·L^−1^ and based on that concentration, grape varieties and fruits can be arranged in order: Zweigelt < Pinot Gris < 400 μg·L^−1^ < Sibera < Chardonnay < Gewürztraminer < Cabernet Cortis < 500 μg·L^−1^ < mixed < Solaris < 600 μg·L^−1^ < Rondo < 700 μg·L^−1^ < fruits < Seyval Blanc < Regent < Riesling < 800 μg·L^−1^ < Pinot Noir < Hibernal.

Grape varieties and fruits, based on their mean concentrations of all toxic metals, can be arranged in order: Sibera < Zweigelt < Rondo < Cabernet Cortis < Regent < Solaris < mixed < Pinot Noir < Gewürztraminer < 1000 μg·L^−1^ < Chardonnay < Riesling < Seyval Blanc < Pinot Gris < 2000 μg·L^−1^ < fruits < Hibernal. Just like for mean concentrations of all toxic metals, the highest mean concentrations of all heavy metals were found for Hibernal wines and fruit wines.

The highest mean metal concentration was obtained for K in wines produced from all grape varieties and fruits. The lowest mean metal concentration was obtained for Hg (Pinot Noir, Riesling, Gewürztraminer, Cabernet Cortis, Chardonnay, Pinot Gris, mixed grape varieties and fruits), and for wine samples in which Hg was not determined, the lowest mean concentrations were obtained for Se (Seyval Blanc, Regent, Sibera, Hibernal and Rondo) and for Cd (Zweigelt and Solaris).

Bekker et al. (2019) analyzed metal ion concentrations in Australian and world Chardonnay wines. Mean concentrations of Cr (16 μg·L^−1^), Fe (1558 μg·L^−1^), Mg (117 mg·L^−1^) and Na (46 mg·L^−1^) were higher in Australian and world Chardonnay wines than in Polish Chardonnay wines. Mean concentrations of Al (388 μg·L^−1^), Cu (36 μg·L^−1^), K (583 mg·L^−1^), Mn (610 μg·L^−1^), Ni (18 μg·L^−1^) and Zn (445 μg·L^−1^) were lower, and concentrations of Ca (59 mg·L^−1^) and Co (6 μg·L^−1^) were similar [21].

Ivić et al. (2021) analyzed elements in conventional and ecological Cabernet Sauvignon wines. In our study, wines from other Cabernet variety grapes were tested—Cabernet Cortis. The mean concentrations (conventional wine and ecological wine) of Mn (1925.6, 1838.2 μg·L^−1^), Fe (1785.0, 1317.8 μg·L^−1^), Cu (447.9, 496.8 μg·L^−1^), Zn (1400.5, 1212.9 μg·L^−1^) and Pb (20.7, 25.8 μg·L^−1^) were higher in Cabernet Sauvignon wines than those analyzed in this study. For Cabernet Cortis wines, the mean concentration of K (597.7, 748.7 mg·L^−1^) was lower, and the mean concentrations of Ca (55.7, 50.7 mg·L^−1^) and Sr (260.6, 520.6 μg·L^−1^) were comparable [16].

Tanabe et al. (2020) analyzed Pinot Noir wines produced in 2015 and 2017 within one American viticultural area. The mean concentrations of Co (5.43 μg·L^−1^), Ba (315 μg·L^−1^), B (6.37 mg·L^−1^), Sr (0.94 mg·L^−1^), Mg (131 mg·L^−1^), Fe (1979 μg·L^−1^) and Sr (0.94 mg·L^−1^) were higher in American Pinot Noir wines than in Polish Pinot Noir wines, mean concentrations of V (0.31 μg·L^−1^), Cd (0.152 μg·L^−1^), Sb (0.06 μg·L^−1^), Pb (3.13 μg·L^−1^), Ca (54.9 mg·L^−1^), K (738 mg·L^−1^), Al (204 μg·L^−1^), Ti (13.7 μg·L^−1^), Cr (3.90 μg·L^−1^), Zr (2.20 μg·L^−1^), Cu (50.3 μg·L^−1^) and As (1.135 μg·L^−1^) were lower and mean concentrations of Li (5.43 μg·L^−1^), Ni (38.0 μg·L^−1^) and Mn (2.18 mg·L^−1^) were comparable [13].

Płotka-Wasylka et al. (2018) analyzed concentrations of seven elements: K (mean values: 301 mg·L^−1^ in fruit wines and 430.5 mg·L^−1^ in grape wines), Ca (24.7 and 18.6 mg·L^−1^), Mg (14.8 and 12.4 mg·L^−1^), Pb (35.5 and 25.9 μg·L^−1^), Zn (133 and 110.5 μg·L^−1^), Cd (3.01 and 0.84 μg·L^−1^) and Fe (0.54 and 1.24 mg·L^−1^) in Polish fruit wines (18 samples) and grape wines (4 samples). The lowest concentration was determined for Cd, both in fruit wines and grape wines, which is similar to the results of our study (0.829 and 0.806 μg·L^−1^) if we take into account only those seven elements that were analyzed in both studies. Contents of Pb and Zn were also low in both studies, but concentrations of Pb were lower in our study (12.82 and 12.16 μg·L^−1^) and concentrations of Zn were higher in our study (290.5 and 675.6 μg·L^−1^). We determined Fe in grape wines (1.426 mg·L^−1^) at a comparable concentration as Płotka-Wasylka et al. (2018), but its concentration in fruit wines was higher in our study (3.670 mg·L^−1^). Mg concentration was determined at similar levels in fruit wines and grape wines in each study, but we determined it at higher concentrations (86.85 and 84.04 mg·L^−1^). Ca was analyzed at comparable concentrations in fruit wines (27.79 mg·L^−1^), but we found it at higher concentrations in grape wines (72.26 mg·L^−1^). Potassium was detected at the highest concentrations in both studies, but we observed it at higher concentration levels (1965 and 1256 mg·L^−1^) [17]. In an earlier study published by Płotka-Wasylka et al. (2017), fruit wines (17 samples) were also analyzed for concentrations of K, Ca, Mg, Pb, Zn, Cd and Fe, as above, and additionally for Hg and Sn. However, Sn was not determined in any of the samples, and Hg was found in only one, at a concentration of 0.437 μg·L^−1^, which is higher than the mean concentration obtained in our study for fruit wine samples (0.107 μg·L^−1^) [35].

### 3.3. Alcohol Content

The alcohol content in the analyzed wines ranged from 8.5 to 14.5%. Wines were divided into four groups based on their content: A: 8.5–9.5%, B: 9.6–11.5%, C: 11.6–13.5% and D: 13.6–14.5%. The highest total of mean concentrations of all metals was obtained for wines with alcohol content in the range of A (2.207 g·L^−1^), and the lowest for wines with alcohol content in the D group (1.374 g·L^−1^). Based on the total mean concentrations of all metals, the alcohol content in wines can be arranged in the following order: group D < group B < group C < group A. The lowest mean metal concentrations were obtained for Hg (for groups A, B and C) and for Se (group D; Hg was not determined in that group). The highest mean metal concentration was obtained for K in all four groups. The highest mean concentrations of all macrominerals—2.195 g·L^−1^, all essential minerals—9.214 mg·L^−1^, all trace metals—727.9 μg·L^−1^, all toxic metals—2304 μg·L^−1^ and all heavy metals—5454 μg·L^−1^—were found in wines of group A. The lowest mean concentrations of all macrominerals—1.367 g·L^−1^, all essential minerals—5962 μg·L^−1^ and all toxic metals—317.6 μg·L^−1^—were found in wines of group D and the lowest mean concentrations of all trace metals—549.9 μg·L^−1^—were found in wines of group B.

### 3.4. Place of Growth and Production

Poland is divided into 16 voivodeships, and the wines analyzed in this study were produced in eight of them: the Lower Silesian Voivodeship, the Subcarpathian Voivodeship, the Silesian Voivodeship, the Lubusz Voivodeship, the Lesser Poland Voivodeship, the Świętokrzyskie (Holy Cross) Voivodeship, the West Pomeranian Voivodeship and the Lublin Voivodeship.

The highest total of mean concentrations of all metals was obtained for wines produced in Lublin Voivodeship (2.207 g·L^−1^) and the lowest for wines produced in Subcarpathian Voivodeship (1.111 g·L^−1^). Based on the highest total mean concentrations of all metals in wine, their origins can be arranged in order: Subcarpathian Voivodeship < 1.2 g·L^−1^ < Lubusz Voivodeship < Lower Silesian Voivodeship < 1.4 g·L^−1^ < Silesian Voivodeship < Świętokrzyskie (Holy Cross) Voivodeship < 1.6 g·L^−1^ < Lesser Poland Voivodeship < West Pomeranian Voivodeship < 2.2 g·L^−1^ < Lublin Voivodeship. The lowest mean metal concentrations were determined for Hg (Lower Silesian Voivodeship, Subcarpathian Voivodeship, Lubusz Voivodeship, Lesser Poland Voivodeship and Lublin Voivodeship) and for Se (Silesian Voivodeship, Świętokrzyskie (Holy Cross) Voivodeship and West Pomeranian Voivodeship), for wines in which Hg was not determined. In wines from all regions, the highest mean metal concentration was obtained for K.

The highest mean concentrations of all macrominerals—2.195 g·L^−1^, all essential minerals—9214 μg·L^−1^, all trace metals—727.9 μg·L^−1^, all toxic metals—2304 μg·L^−1^ and the second highest mean concentration of all heavy metals—5454 μg·L^−1^ were obtained for wines from Lublin Voivodeship. The highest mean concentration of all heavy metals was obtained for wines from Świętokrzyskie (Holy Cross) Voivodeship—5762 μg·L^−1^. The lowest mean concentrations of all macrominerals—1.102 g·L^−1^ and all heavy metals—3236 μg·L^−1^ was found in wines from Subcarpathian Voivodeship, with the lowest mean concentration of all essential minerals—5665 μg·L^−1^ were found in wines from West Pomeranian Voivodeship; the lowest mean concentration of all trace metals—507.9 μg·L^−1^ were found in wines from Lubusz Voivodeship; and the lowest mean concentration of all toxic metals—302.4 μg·L^−1^ were found in wines from Silesian Voivodeship.

It is worth emphasizing that the results of the analyses showed characteristic concentration trends for selected elements (Table S4). Among the 26 analyzed elements, without taking macroelements into account, it was observed that Fe, Mn and B were determined at levels above 1000 µg·L^−1^ in all analyzed wines from different voivodeships. Moreover, characteristic trends of increasing concentrations between vineyards in voivodeships were observed. Additionally, for four voivodeships (Lower Silesian, Subcarpathian, Lesser Poland and West Pomeranian), there was an upward trend of Fe > Mn > B, for the remaining three (Silesian, Lubusz and Lublin), Mn > Fe > B; and for Świętokrzyskie (Holy Cross), Mn > B > Fe. The obtained results can be a kind of fingerprint for the analyzed wines from a given vineyard in a particular voivodeship.

Alkıs et al. (2014) analyzed the content of Cr, Mn, Fe, Co, Ni, Cu, Zn, Cd and Pb in Turkish white and red wines from four different regions—Aegean, Marmara, Central Anatolia and Eastern Anatolia. Concentrations of analyzed elements were quite similar in all four regions; nonetheless, the biggest differences in content between regions were observed for manganese, with relatively high values of its mean concentration in wines from Central Anatolia and Eastern Anatolia, especially in red wines [36].

Research conducted by Cepo et al. (2022) on Croatian Continental and Adriatic red and white wines showed that K, Mn, Ba and Ni can be used to discriminate between continental red and white wines and that Rb, Ni and Ba can be used to discriminate between Continental red and Adriatic red wines. Sr can be used to discriminate between all four wine groups [14].

Hao et al. (2021) analyzed elemental (K, Ca, Mg, Na, Fe, Rb, Cu, Sr, Mn, Zn, Al, Ba, Li, Cs and Cd) profiles of wines in six different Chinese regions: Yangling, Bailuyuan, Heyang, Xiaxian, Minquan and Wuhai. The highest concentrations of Mn, Zn and Sr were found in wines from Xiaxian. The lowest concentrations of Fe, Zn, Sr, Li and Ba were obtained in wines from Bailuyuan, and the lowest concentrations of Mn, Rb, Cd and Cs were found in wines from Wuhai [19].

In our research, boron was determined to have the highest range of concentrations in all wine samples compared to other elements, not including macronutrients. This may indicate that B is a specific marker characteristic of Polish wines. Gajek et al. (2021), based on the highest median values, showed that such markers for the analyzed wines from Australia were Mn and Sr, and for wines from the USA it was U. In both cases, high, country-specific concentrations were associated with the type of soil, including naturally occurring mineral resources or the climatic conditions of the wine-growing area [24].

### 3.5. Health Risk Assessment

In this study, ADI (Average Daily Intake) values were determined for all tested elements. THQ (Target Hazard Quotient) and HI (Hazard Index) values were determined for Al, As, B, Ba, Be, Cd, Cr, Cu, Hg, Mn, Ni, Pb, Sb, Se, Sr, V and Zn (Table 2 and Table 3), and CR (carcinogenic risk) values were determined for As, Cd and Pb (Table 2 and Table 3), and were calculated for average daily consumption of wine by Polish people; in 2021, yearly consumption of wine and mead by a Polish person was 6.7 liters [2] (Table 2), and for a glass of wine (150 mL, Table 3), both calculated for 70 kg of body weight.

#### 3.5.1. Average Daily Consumption of Wine by Polish People

Total ADI values for concentrations of all elements per wine ranged from 87.82 μg·kg^−1^·day^−1^ (no. 28B) to 962.8 μg·kg^−1^·day^−1^ (no. 3-B), with a mean value of 390.0 μg·kg^−1^·day^−1^. Analyzed metals, based on their mean ADI values, can be arranged in order: Hg < 10^−4^ μg·kg^−1^·day^−1^ < Se < Sb < Cd < Be < Li < Co < As < 10^−3^ μg·kg^−1^·day^−1^ < Pb < Cr < V < 0.01 μg·kg^−1^·day^−1^ < Ni < Zr < Cu < Ti < Ba < 0.1 μg·kg^−1^·day^−1^ < Sr < Zn < Al < Fe < Mn < 1 μg·kg^−1^·day^−1^ < B < Na < 10 μg·kg^−1^·day^−1^ < Ca < Mg < 100 μg·kg^−1^·day^−1^ < K. THQ values were calculated for Al, As, B, Ba, Be, Cd, Cr, Cu, Hg, Mn, Ni, Pb, Sb, Se, Sr, V and Zn. Obtained mean THQ values can be arranged in order: Cr < Se < Al < 10^−4^ < Sr < Ba < Hg < Cd < Be < Sb < Cu < Zn < V < Ni < Pb < 10^−3^ < As < Mn < B, and the accumulative value of HI was 0.016. HI values ranged from 0.013 (no. 4B) to 0.015 (no. 3-B), with a mean value of 0.015. CR values were calculated for As, Cd and Pb, and the mean CR values can be arranged in the order of Pb < Cd < As (Table 2 and Table 4).

#### 3.5.2. For One Glass of Wine—150 mL

Total ADI values for concentrations of all elements per wine ranged from 717.7 μg·kg^−1^·day^−1^ (no. 28B) to 7868 μg·kg^−1^·day^−1^ (no. 3-B), with a mean value of 3187 μg·kg^−1^·day^−1^. The mean ADI values for each element in all wines ranged from 1.559·10^−4^ μg·kg^−1^·day^−1^ (Hg) to 2807 μg·kg^−1^·day^−1^ (K). The mean THQ values for each element in all wines ranged from 2.097·10^−5^ (Cr) to 4.138·10^−2^ (B), with an accumulative HI value of 0.129. Obtained HI values ranged from 0.055 (no. 4B) to 0.271 (no. 3-B), with a mean value of 0.125. CR values were calculated for As, Cd and Pb, and the mean CR values can be arranged in the order Pb < Cd < As (Table 3 and Table 4).

In white wines, elements, based on their mean ADI values, can be arranged in order: Hg < Se < Cd < Sb < Be < Li < Co < As < Cr < Pb < V < Ni < Zr < Cu < Ti < Ba < Sr < Zn < Al < Mn < Fe < B < Na < Ca < Mg < K. In red wines, elements, based on their mean ADI values, can be arranged in order: Hg < Be < Se < Sb < Cd < Li < As < Co < Pb < V < Cr < Zr < Ni < Cu < Ti < Ba < Al < Sr < Zn < Fe < Mn < B < Na < Ca < Mg < K. In white wines, elements, based on their mean THQ values, can be arranged in order: Cr < Se < Al < Sr < Ba < Cd < Hg < Be < Sb < Zn < Ni < Cu < V < Pb < Mn < As < B. In red wines, elements, based on their mean THQ values, can be arranged in order: Cr < Be < Al < Se < Hg < Ba < Sr < Cd < Sb < Cu < V < Zn < Ni < Pb < As < Mn < B.

On the basis of the results obtained for individual grape varieties, ADI and THQ were calculated. It was observed that for all wine varieties, the highest ADI value was determined for potassium and the lowest for mercury, selenium and cadmium. In the case of THQ, it was observed that for as many as 11 analyzed grape varieties, the highest values were determined for boron (max. Pinot Gris variety; THQ = 0.011). It is also worth noting that for the varieties Hibernal (THQ = 0.010), Resling (THQ = 0.006) and Syval Blanc (THQ = 0.004), the maximum values of this indicator were determined for arsenic, whose highest values were comparable to the highest values for boron, despite significant differences in the aromas of these grape varieties. While the highest HI values were determined for the Hibernal variety (HI = 0.025) and the lowest for the Sibera variety (HI = 0.008). It should be emphasized that these are strains that differ in aromas; the Sibera strain has mainly citrus aromas, and the Hibernal strain has the aromas of tropical fruits or flowers.

All THQ values were below 1, which indicates that there is no risk to human health during daily consumption of wine at the determined levels of metal concentrations. All HI values were lower than 1, which indicates minimal exposure to risk and no significant health risk for people consuming wine. All determined CR values were below or in the permissible range for carcinogenic risk over a lifetime of 70 years, as recommended by USEPA, at levels from 10^−6^ to 10^−4^.

Deng et al. (2019) determined THQ values for arsenic (0.069), cadmium (0.002), manganese (0.078), nickel (0.007), zinc (0.005), selenium (0.006) and chromium (0.159) in wine (200 mL wine, 60 kg of body weight). Mean THQ values for cadmium and zinc were comparable to THQ values obtained in Polish wines, and mean THQ values for arsenic, manganese, nickel, selenium and chromium were higher in Chinese wines than in Polish wines [11].

Cepo et al. (2020) obtained THQ values for copper, iron, manganese, nickel, zinc, lead and chromium in Continental and Adriatic white and red wines (6 g of wine per day, 63/86 kg of male/female body weight). All obtained THQ values were below 0.0020 and were similar to THQ values obtained in this study (18.356 mL of wine per day, 70 kg of body weight), with THQ values of copper, manganese, nickel, zinc and lead being slightly higher in Polish wines and with THQ values of chromium being slightly lower in Polish wines. Daily consumption of wine in Croatia was lower than in Poland [14].

In our study, THQ values were calculated for seventeen metals, which is more than in the presented articles. Referring to the aspect related to the nutritional value of analyzed elements in wine, it is assumed that the contribution of wine to the total dietary intake of particular nutritional metals is usually up to maximally 2–3% [14]. In spite of the good aspects of wine, we should also remember that it can be a source of potentially toxic metals in human nutrition. That is why in this study we estimated the potential human risk by calculating various factors and making a comparison with the permissible levels of particular elements in wine. Consequently, the content of the analyzed elements in this study was compared with the maximum permissible values at the international level (Office International de la Vigne et du Vin, O.I.V., standards (Organization Internationale de la Vinge et du Vin, O.I.V., 2008). The comparison showed that the maximum permissible concentration values given by O.I.V. for the following elements: Ni, Cu, Zn, Cr, Pb and Fe in white wine and Fe in red wine (0.1; 1.0; 5.0; 0.1; 0.15; 10.0 and 20.0, respectively) [14] were not exceeded in the analyzed Polish wines. Only in the case of three white wines and one red wine were the maximum values above the allowed value for nickel. However, taking into account the average values, the limits for nickel have not been exceeded.

### 3.6. Chemometrics Analysis

**Figure 2:**
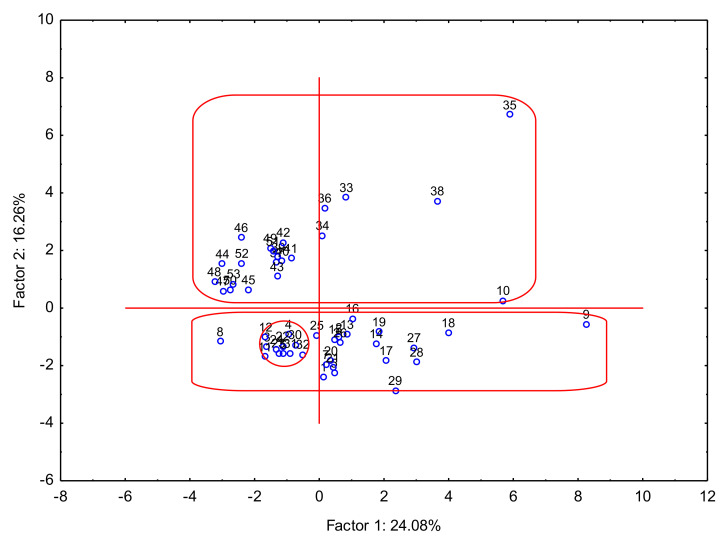
Principal Component Analysis of 53 wine samples from Polish vineries.

**Figure 3:**
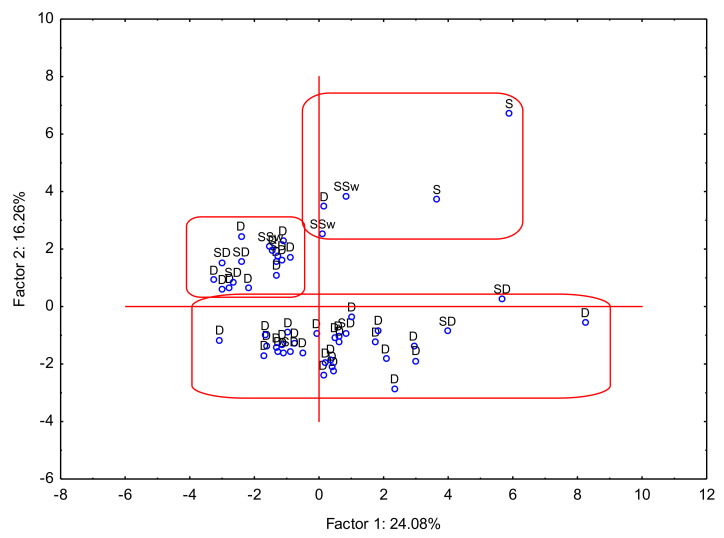
Principal Component Analysis PC1. vs. PC2 for dryness of wine.

The analysis of the red and white wines was performed for all elements and alcohol contents. The variance for the two factors was 24.08% and 16.26%, respectively, which gives a total value of 40.34%. The Principal Component Analysis for the difference between sample compositions based on the analysis of all elements is presented in Figure 2. Description of wine samples: D—Dry; SD—Semi Dry; SSw—Semisweet and S—sweet, wine strains (PG—Pinot Gris; Zw—Zweigelt; Ge—Gewürztraminer; Che—Cherries; Rib—Ribes nigrum (blackcurrant); PN—Pinot Noir; Ch—Chardonnay; SB—Seyval Black; Si—Sibera; Bi—Bianca Re—Regent; Cb—Cabernet Cortis; Ro—Rondo; Ris—Riesling; So—Solaris; Jo—Johanniter Hi—Hiberal). Taking into account the results from the PCA, three clusters might be designated: (1) wines from Lower Silesia Voivodeship (Dolnośląskie), Subcarpathia Voivodeship (Podkarpackie), Silesia Voivodeship (Śląskie) and Lubusz (Lubuskie); (2) Holy Cross Voivodeship (Świętokrzyskie), West Pomerania Voivodeship (Zachodniopomorskie), Lesser Poland Voivodeship (Małopolskie) and Lublin Voivodeship (Lubelskie); and (3) Kraków, Lesser Poland Voivodeship (Kraków, Małopolskie). Based on the results, it was observed that wines can be classified based on the place of their cultivation, but it was also confirmed that the type of wine played a fundamental role in its classification (Figure 3). It should be concluded that similarities and differences in wine composition were associated with the type of wine. The first group was represented by D wine, the second group by D and SD, and the last, quite scattered, group by SSw and S.

**Figure 4:**
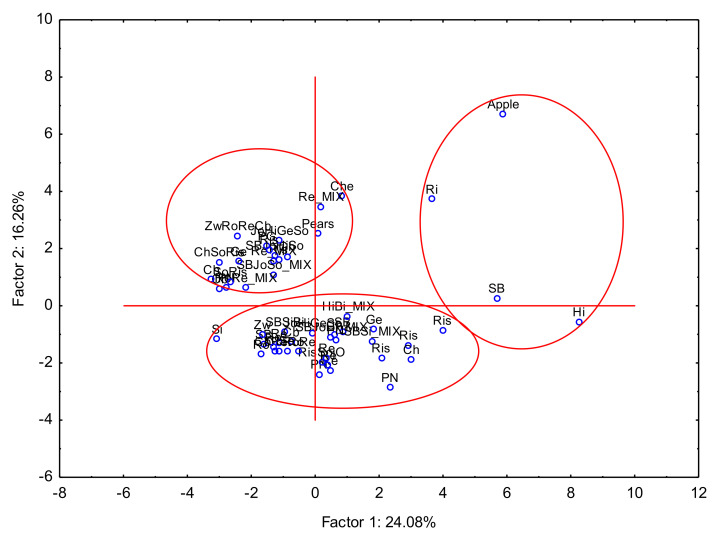
Principal Component Analysis PC1. vs. PC2 for wine strain.

The next important issue was to demonstrate the wine strain similarities based on PCA analysis. The results of the PCA analysis are presented in Figure 4, and this analysis could not be fully defined because, based on its results, it could be concluded that the grape variety did not define the type of wine due to its elemental composition.

**Figure 5:**
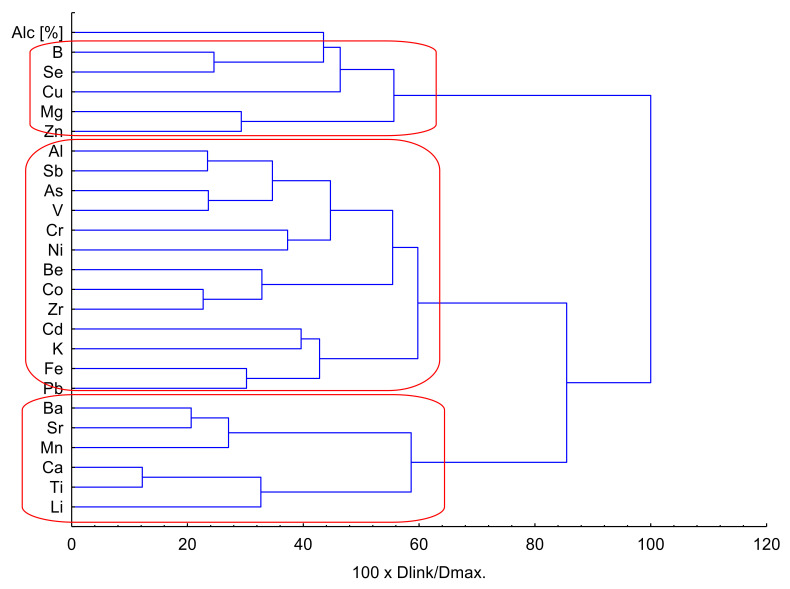
Hierarchical dendrogram for 25 variables.

Next, after PCA analysis, Hierarchical Cluster Analysis (HCA) was performed after z-transformation of the input raw data (Ward’s method of linkage) and cluster significance by Sneath’s index (Figure 5).

Analysis showed the formation of three main clusters: K1—Li, Ti, Ca, Mn, Sr, Ba; K2—Pb, Fe, K, Cd, Zr, Co, Be, Ni, Cr, V, As, Sb, Al; and K3—Zn, Mg, Cu, Se, B, Alc [%].

After clustering with the use of HCA, the factor analysis was performed and presented in Table 5. To obtain more than 80% of the total variance, eight principal components were selected in this analysis. Factor 1 explained 24.08% of the total variance and was connected with the high correlation between Al, Be, Co and Zr, which was related to K2 in HCA. Factor 2 explained 16.26% of total variance and corresponded to K1 (Ti and Ca) and K3 (B and Se) in HCA. It should be stressed here that B and Se were negatively correlated in the K3 cluster. It might be a reason why those elements were in two different clusters. Factor 3 explained 10.08% of the total variance and included Mg and Zn (K3 in HCA), both of which were negatively correlated. Next Factor 4 explained 8.18% of total variance, showed correlation between Ba, Mn and Sr, and represented the K1 cluster in HCA. The last Factors 5–8 explained 7.18%, 6.17%, 4.97% and 3.99%, respectively, and were correlated in Factor 5 with Fe, in Factor 6 with Cr and V and in Factor 7 with Pb. All factor loadings for Fe, Cr, V and Pb corresponded to K2 in HCA. Moreover, Factor 8 was negatively correlated with Cu. Factor analysis showed that both analyses, FA and HCA, had similar relationships, and it should be concluded here that K1 and K3 were closely related to local conditions and soil properties, and, in this case, it might suggest that for Cu and its negative correlation, this could indicate the type of water. Anyway, the K2 cluster and FA showed correlation, which might be caused by the soil contamination (Fe, Cr, V and Pb). On the other side, the impact of other toxic elements, represented by K2, was not observed.

**Figure 6:**
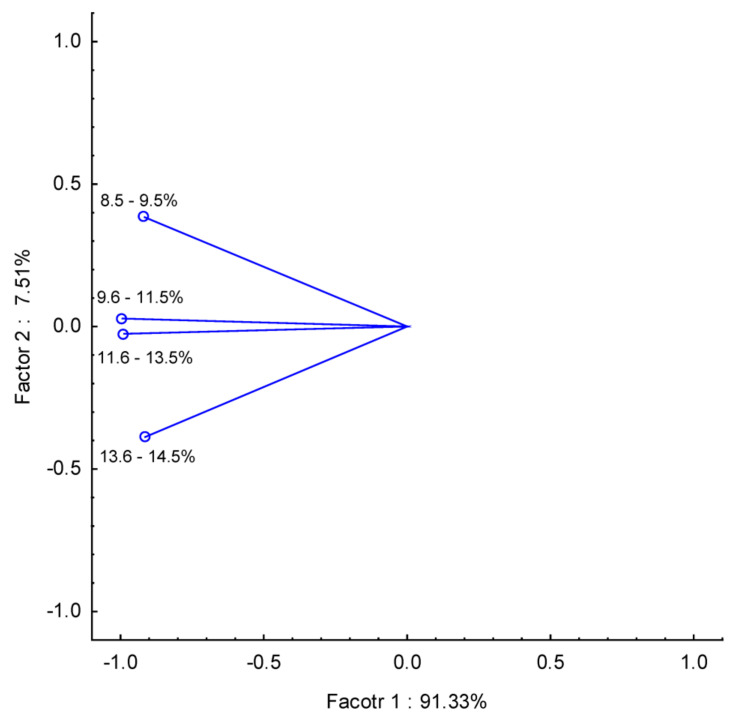
PCA for % of alcohol vs. THQ.

**Figure 7:**
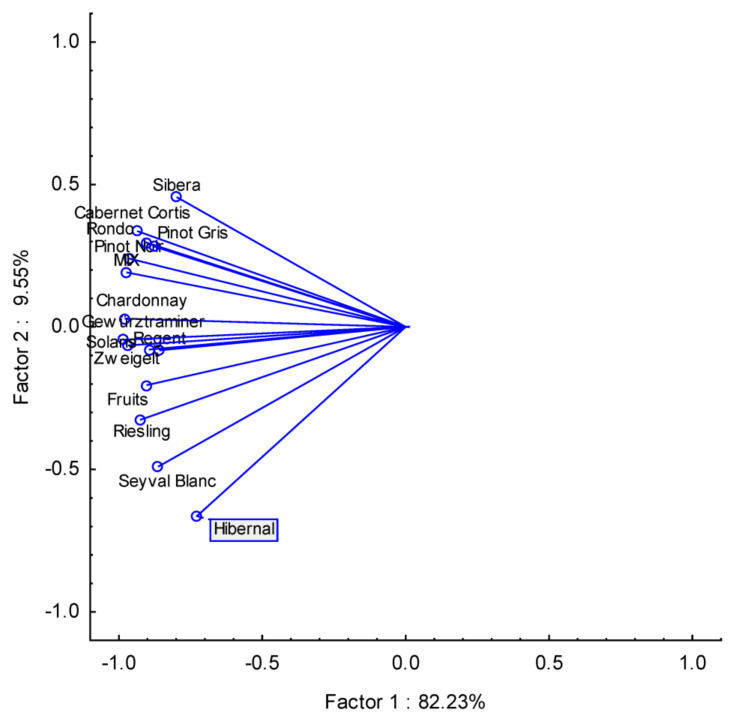
PCA of THQ vs. wine grape varieties.

As a new approach, we applied chemometric tools to visually present potential correlation in health risk among analyzed wines according to their alcohol content and grape varieties. PCA for % of alcohol and THQ data showed a correlation between wines with 9.6–11.5% and 11.6–13.5% of alcohol and a slightly weaker correlation but still strong between 8.5 and 9.5% and 13.6–14.5% in PC1. Based on this PCA data analysis, it can be concluded that alcohol content did not affect the THQ index. Similar data interpretation can be found for PCA results between wine strain and THQ index (Figure 6 and Figure 7).

## 4. Conclusions

In this study, 53 Polish wines from eight regions of Poland were subjected to multielement analysis. The highest mean concentrations of macrominerals, essential minerals, trace and toxic metals were obtained in wines from Lublin Voivodeship (2.195 g·L^−1^, 9214 μg·L^−1^, 727.9 μg·L^−1^ and 2304 μg·L^−1^, respectively). The highest mean concentration of heavy metals (5762 μg·L^−1^) was obtained in wines from Świętokrzyskie (Holy Cross) Voivodeship and the lowest mean concentration (3236 μg·L^−1^) was found in wines from Subcarpathian Voivodeship. While the lowest mean concentrations of macrominerals, essential minerals, trace and toxic metals were found in wines from Subcarpathian Voivodeship, Pomeranian Voivodeship, Lubusz Voivodeship and Silesian Voivodeship (1.102 g·L^−1^, 5665 μg·L^−1^, 507.9 μg·L^−1^ and 302.4 μg·L^−1^, respectively).

For all the analyzed wines, the highest concentrations of macronutrients were determined for potassium (range 0.120–3.310 g·L^−1^). On the other hand, for the rest of the elements, excluding macronutrients, the highest concentrations in both red and white wines were determined for boron (range 1870–9080 mg·L^−1^, 1400–8550 mg·L^−1^, respectively), which may be a specific factor characterizing Polish wines, taking into account the literature data for wines from other regions of the world, where much lower concentrations of this nutrition element were determined.

The data obtained from the health risk assessment showed that none of the wine samples exceeded the toxic levels of metals reported in the literature. This was confirmed by the estimation of the daily intake of a given element and THQ values, which did not exceed 1 for any element in the analyzed wines (THQ < 1).

The analysis of PCA was applied to the data on the elemental composition and revealed a distinction between three groups of wines depending on geographical origin. In addition, the PCA analysis made it possible to distinguish three characteristic categories of wines depending on the degree of dryness of the analyzed wines. On the other hand, the HCA analysis distinguished three clusters, for which the factor analysis made it possible to observe positive and negative correlations between individual elements and the main HCA clusters. The chemometric analysis confirmed that elements such as Li, Ti, Ca, Mn, Sr, Ba, Zn, Mg, Cu, Se and B were closely related to local conditions and soil properties, and the presence of Fe, Cr, V and Pb was related to contamination of the soil.

## Figures and Tables

**Table 1:** Characteristic conditions for analytics parameters of ICP-MS and ICP-OES spectrometers.

Parameters and Accessories	ICP-MS	ICP-OES
Radio frequency power generator [kW]	1.2	1.2
Gas type	Argon	Argon
Plasma gas flow rate [L·min^−1^]	8.0	9.0
Auxiliary gas flow rate [L·min^−1^]	1.1	0.6
Nebulization gas flow rate [L·min^−1^]	0.7	0.7
Torch	Mini torch (quartz)	Mini torch (quartz)
Nebulizer	Coaxial	Coaxial
Spray chamber temperature	3 °C	Room temperature
Drain	Gravity fed	Gravity fed
Internal standard	Automatic addition	–
Sampling depth	5 mm	–
Collision cell gas flow (He)	6.5 mL·min^−1^	–
Cell voltage	–21 V	–
Energy Filter	7.0 V	–
Number of replicates	3	3
Integration conditions/number of scans	10	–

**Table 2:** Estimation of selected elements: average dietary intake (ADI) [in µg/kg of body weight/day], target hazard quotient (THQ), carcinogenic risk (CR) and hazard index (HI) for all analyzed wine samples. RfD [µg/kg of body weight/day].

Per Daily Intake Calculated from Average Yearly Consumption in Poland										
Element	RfD	All Wine Samples (N = 53)			Red Wine Samples (N = 22)			White Wine Samples (N = 31)		
		ADI	THQ	CR	ADI	THQ	CR	ADI	THQ	CR
Al	7000	0.171	2.441·10^−5^		0.108	1.543·10^−5^		0.215	3.078·10^−5^	
As	0.3	8.930·10^−4^	2.977·10^−3^	1.340·10^−6^	5.684·10^−4^	1.895·10^−3^	8.526·10^−7^	1.123·10^−3^	3.745·10^−3^	1.685·10^−6^
B	200	1.013	5.064·10^−3^		1.011	5.055·10^−3^		1.014	5.070·10^−3^	
Ba	200	0.037	1.867·10^−4^		0.043	2.141·10^−4^		0.033	1.672·10^−4^	
Be	2	4.674·10^−4^	2.337·10^−4^		2.318·10^−5^	1.159·10^−5^		5.357·10^−4^	2.679·10^−4^	
Cd	1	2.118·10^−4^	2.118·10^−4^	8.048·10^−8^	2.447·10^−4^	2.447·10^−4^	9.298·10^−8^	1.905·10^−4^	1.905·10^−4^	7.241·10^−8^
Cr	1500	3.849·10^−3^	2.566·10^−6^		4.120·10^−3^	2.747·10^−6^		3.657·10^−3^	2.438·10^−6^	
Cu	40	0.022	5.549·10^−4^		0.013	3.148·10^−4^		0.029	7.238·10^−4^	
Hg	0.1	1.908·10^−5^	1.908·10^−4^		1.813·10^−5^	1.813·10^−4^		1.967·10^−5^	1.967·10^−4^	
Mn	140	0.419	2.995·10^−3^		0.480	3.431·10^−3^		0.376	2.684·10^−3^	
Ni	20	0.012	6.220·10^−4^		0.011	5.447·10^−4^		0.014	6.769·10^−4^	
Pb	3.5	3.202·10^−3^	9.149·10^−4^	2.722·10^−8^	2.476·10^−3^	7.075·10^−4^	2.105·10^−8^	3.717·10^−3^	1.062·10^−3^	3.160·10^−8^
Sb	0.4	1.630·10^−4^	4.074·10^−4^		1.202·10^−4^	3.006·10^−4^		1.933·10^−4^	4.832·10^−4^	
Se	5	1.113·10^−4^	2.226·10^−5^		1.176·10^−4^	2.352·10^−5^		1.068·10^−4^	2.136·10^−5^	
Sr	600	0.109	1.825·10^−4^		0.130	2.165·10^−4^		0.095	1.584·10^−4^	
V	9	5.247·10^−3^	5.829·10^−4^		2.934·10^−3^	3.261·10^−4^		6.813·10^−3^	7.570·10^−4^	
Zn	300	0.170	5.651·10^−4^		0.159	5.304·10^−4^		0.177	5.897·10^−4^	
**HI**			0.016			0.014			0.017	

RfD values for Al, As, B, Ba, Be, Cd, Cr, Cu, Mn, Ni, Sb, Se, Sr, V and Zn are given by EPA’s Integrated Risk Information System (IRIS) [37], and RfD values for Hg and Pb are taken from [22]. Used CSF values for calculation of CR: Pb 85·10^−4^, Cd 38·10^−2^, As 15·10^−1^ and Hg 0 in (mg kg^−1^ day^−1^)^−1^ [22].

**Table 3:** Estimation of selected elements: average dietary intake (ADI) [in µg/kg of body weight/day], target hazard quotient (THQ), carcinogenic risk (CR) and hazard index (HI) for all analyzed wine samples per glass of each type of wine. RfD [µg/kg of body weight/day].

Per Glass of Wine (150 mL)										
Element	RfD	All Wine Samples (N = 53)			Red Wine Samples (N = 22)			White Wine Samples (N = 31)		
		ADI	THQ	CR	ADI	THQ	CR	ADI	THQ	CR
Al	7000	1.396	1.995·10^−4^		0.883	1.261·10^−4^		1.761	2.515·10^−4^	
As	0.3	7.297·10^−3^	2.432·10^−2^	1.095·10^−5^	4.645·10^−3^	1.548·10^−2^	6.967·10^−6^	9.180·10^−3^	3.060·10^−2^	1.377·10^−5^
B	200	8.275	4.138·10^−2^		8.262	4.131·10^−2^		8.285	4.143·10^−2^	
Ba	200	0.305	1.525·10^−3^		0.350	1.750·10^−3^		0.273	1.366·10^−3^	
Be	2	3.819·10^−3^	1.910·10^−3^		1.894·10^−4^	9.471·10^−5^		4.378·10^−3^	2.189·10^−3^	
Cd	1	1.731·10^−3^	1.731·10^−3^	6.576·10^−7^	2.000·10^−3^	2.000·10^−3^	7.598·10^−7^	1.557·10^−3^	1.557·10^−3^	5.917·10^−7^
Cr	1500	0.031	2.097·10^−5^		0.034	2.244·10^−5^		0.030	1.992·10^−5^	
Cu	40	0.181	4.534·10^−3^		0.103	2.573·10^−3^		0.237	5.914·10^−3^	
Hg	0.1	1.559·10^−4^	1.559·10^−3^		1.481·10^−4^	1.481·10^−3^		1.607·10^−4^	1.607·10^−3^	
Mn	140	3.426	2.447·10^−2^		3.926	2.804·10^−2^		3.071	2.194·10^−2^	
Ni	20	0.102	5.083·10^−3^		0.089	4.451·10^−3^		0.111	5.531·10^−3^	
Pb	3.5	0.026	7.476·10^−3^	2.224·10^−7^	0.020	5.781·10^−3^	1.720·10^−7^	0.030	8.679·10^−3^	2.582·10^−7^
Sb	0.4	1.332·10^−3^	3.329·10^−3^		9.826·10^−4^	2.456·10^−3^		1.579·10^−3^	3.949·10^−3^	
Se	5	9.096·10^−4^	1.819·10^−4^		9.609·10^−4^	1.922·10^−4^		8.727·10^−4^	1.745·10^−4^	
Sr	600	0.895	1.491·10^−3^		1.062	1.769·10^−3^		0.776	1.294·10^−3^	
V	9	0.043	4.764·10^−3^		0.024	2.664·10^−3^		0.056	6.186·10^−3^	
Zn	300	1.385	4.618·10^−3^		1.300	4.334·10^−3^		1.446	4.819·10^−3^	
**HI**			0.129			0.114			0.137	

RfD values for Al, As, B, Ba, Be, Cd, Cr, Cu, Mn, Ni, Sb, Se, Sr, V and Zn are given by EPA’s Integrated Risk Information System (IRIS) [37], and RfD values for Hg and Pb are taken from [22]. Used CSF values for calculation of CR: Pb 85·10^−4^, Cd 38·10^−2^, As 15·10^−1^ and Hg 0 in (mg kg^−1^ day^−1^)^−1^ [22].

**Table 4:** Estimation of selected elements average dietary intake (ADI) [in µg/kg of body weight/day] for elements that did not have a THQ value calculated.

	Per Daily Intake Calculated from Average Yearly Consumption in Poland			Per Glass of Wine (150 mL)		
Element	ADI [µg/kg of Body Weight/Day]					
	All Wines (N = 53)	Red Wines (N = 22)	White Wines (N = 31)	All Wines (N = 53)	Red Wines (N = 22)	White Wines (N = 31)
Ca	18.07	15.26	20.06	147.6	124.7	163.9
Co	8.105·10^−4^	6.165·10^−4^	9.482·10^−4^	6.623·10^−3^	5.038·10^−3^	7.749·10^−3^
Fe	0.418	0.349	0.468	3.419	2.848	3.824
K	343.5	398.9	304.2	2807	3260	2486
Li	7.028·10^−4^	5.142·10^−4^	8.495·10^−4^	5.743·10^−3^	4.202·10^−3^	6.942·10^−3^
Mg	22.09	21.30	22.66	180.5	174.1	185.1
Na	9.754	8.685	10.41	79.71	70.97	85.09
Ti	0.027	0.020	0.031	0.218	0.163	0.256
Zr	0.015	0.010	0.018	0.125	0.085	0.147

**Table 5:** Factor loading data for 24 variables.

Variable	Factor 1	Factor 2	Factor 3	Factor 4	Factor 5	Factor 6	Factor 7	Factor 8
Al	0.762103	−0.210235	0.109806	0.161249	0.221412	0.247411	0.197132	0.004486
As	0.651838	0.052371	0.031705	0.216722	−0.182407	0.526684	0.256353	0.015705
B	0.042696	−0.865899	−0.095983	−0.099972	−0.118319	−0.003334	0.209572	0.023741
Ba	0.227082	0.064387	0.077892	0.861475	0.137672	0.148543	0.019219	0.083191
Be	0.741863	0.385381	0.089618	0.166290	−0.194549	−0.181065	0.042547	−0.063568
Ca	0.200935	0.821901	−0.082015	0.256170	0.022659	0.043480	0.272000	0.091691
Cd	0.180178	−0.041014	−0.581444	0.001229	0.386675	−0.125185	0.023468	0.182551
Co	0.904969	0.000904	−0.173104	0.064924	0.080499	−0.027878	0.066437	0.070169
Cr	0.027827	0.007206	0.083674	0.065641	0.201250	0.710166	−0.297905	0.012641
Cu	−0.061127	−0.127919	0.019420	−0.108551	−0.056517	−0.104885	0.080157	−0.889427
Fe	0.314450	0.003807	0.081617	−0.103685	0.771825	0.135031	0.312098	0.010155
K	−0.186247	−0.028182	−0.037248	0.390497	0.553530	0.310914	0.095742	0.310032
Li	0.115722	0.556115	−0.014217	0.280608	−0.087460	−0.130657	0.418943	−0.205286
Mg	−0.078651	−0.231446	−0.787875	−0.170914	−0.023490	−0.012971	−0.056864	0.032219
Mn	0.018690	0.166037	−0.000622	0.829702	−0.026435	0.048041	−0.091551	−0.021964
Ni	−0.127860	0.274139	0.069685	0.041656	−0.449022	0.661728	0.102786	0.161022
Pb	0.132949	0.196867	0.012200	−0.032179	0.302228	0.115295	0.853568	−0.063199
Sb	0.373341	−0.212594	0.071433	0.081191	0.404478	0.656923	0.120856	−0.017422
Se	0.251620	−0.782574	−0.176432	0.050893	0.158922	−0.021031	0.017048	−0.143641
Sr	0.078629	0.227780	0.034434	0.883974	−0.054310	0.020811	0.096089	0.066821
Ti	0.282995	0.810016	−0.064558	0.285910	−0.021301	0.060505	0.300729	0.080545
V	0.198484	−0.005293	0.033202	0.080331	0.084296	0.904378	0.174811	0.055202
Zn	−0.094350	0.135616	−0.850656	0.079422	−0.145133	−0.069542	0.030069	−0.122146
Zr	0.806615	0.058878	0.134147	−0.069778	0.265636	0.272897	−0.113777	0.046154

## Data Availability

The data used to support the findings of this study can be made available by the corresponding author upon request.

## Supplementary Materials

The following supporting information can be downloaded at: https://www.mdpi.com/article/10.3390/foods12173205/s1, Table S1: Characterization of wine samples; Table S2: Calibration curve parameters, LOD and LOQ values of ICP-MS/ICP-OES analytical techniques; Table S3: Summary of data for all investigated elements according to the type of wine; Table S4: Minimum and maximum concentrations of elements, mean and median of determined concentrations, analyzed in wine samples depending on their origin—voivodeship.
