# Supplementary material for: Determination of Metal Content by Inductively Coupled Plasma-Mass Spectrometry in Polish Red and White Wine Samples in Relation to Their Type, Origin, Grape Variety and Health Risk Assessment
Source: Foods. 2023 Aug 25;12(17):3205. doi: 10.3390/foods12173205 (PMC10486598; doi:10.3390/foods12173205)
Supplement: Supplementary file 1 [file foods-12-03205-s001.zip › foods-2566402-supplementary.pdf]

foods

MDPI

Table S1. Characterization of wine samples.

No.*	Name	Grape variety	Aroma	Sweet-ness
1A	Pinore XII	Pinot noir	Barrel notes, cherries, rowanberries, cranberries	Dry
2B	Cha-re XIII	Chardonnay, Auxerrois	Tropical fruits, fruit trees	Dry
3B	Cymbały	Seyval Blanc	Citrus fruits, tropical fruits	Dry
4B	Cytryn	Seyval Blanc, Sibera, Bianca	Citrus fruits, herbal notes, tropical fruits	Dry
5A	Koral	Regent	Fruit trees, forest fruits, red fruits, (black) pepper	Dry
6A	Magnesia Prestige	Cabernet Dorsa, Zweigelt, Rondo, Regent	Forest fruits, red fruits, paprika, smoked plum, tobacco	Dry
7B	Passage Cuvee	Riesling, Solaris, Johanniter	Fruit trees, tropical fruits, honey, flower notes	Dry
8B	Sibera	Sibera	Citrus notes, flower notes	Dry
9B	Hibernal	Hibernal	Flower notes, tropical fruits, fruit trees	Dry
10B	Seyval Blanc	Seyval Blanc	Citrus notes, tropical fruits, fruit trees	Semi-dry
11A	Rondo	Rondo	Forest fruits, red fruits, chocolate	Dry
12A	Geltrus XIV	Zweigelt	Forest fruits, red fruits	Dry
13B	Sey Sey XIV	Seyval Blanc	Tropical fruits, nettle leaf	Semi-dry
14B	Lumini XIII	Seyval Blanc, Sibera, Cserszegi Fuseresz, Traminer	Forest fruits, tropical fruits, citrus notes, nettle leaf	Dry
15B	Daromini Blanc	Seyval Blanc, Johaniter, Hiber-nal, Bianka, Sibera	Citrus fruits, tropical fruits, forest fruits	Dry
16B	Hibia XIV	Hibernal, Bianka, Traminer, Cserszegi Fiuszeres	Tropical fruits, citrus notes, flower notes, acidicity	Dry
17B	Parus A	Riesling	Sands, gravel, bog iron ore	Dry
18B	Parus B	Riesling	Sands, gravel, bog iron ore	Semi-dry
19B	Milvus	Gewuertztraminer	Spicy	Dry
20A	Regulus A	Regent	Fruits	Dry
21A	Regulus B	Regent	Fruits	Dry

22A	Regent	Regent	Red fruits, forest fruits, fruit trees, forest litter, compote of dried fruits	Dry
23A	Cabernet Cortis	Cabernet Cortis	Tropical fruit, forest fruits, pepper, red paprika, barrel notes, leather, tobacco, red fruits, fruit trees	Dry
24A	Rondo	Rondo	Red fruits, forrest fruits	Dry
25B	Cuvée Vineyard Srebrna Góra	Johanniter, Hibernal, Gewürztraminer, Solaris	Citrus notes, fruit trees, flower notes, tropical fruits, acidicity	Dry
26A	Pinot Noir	Pinot Noir	Red fruits, spicy notes, acidicity	Dry
27B	Riesling	Riesling	Citrus notes, honey, raisins	Dry
28B	Chardonnay	Chardonnay	Fruits	Dry
29A	Pinot Noir	Pinot Noir	Elderberry, herbal, red fruits, barrel notes	Dry
30A	Cabernet	Cabernet Cortis	Red fruits, fresh pepper, cacao, dark chocolate	Dry
31B	Solaris	Solaris	Tropical fruits, fruit trees, gooseberry	Semi-dry
32A	Rondo/Reget	Rondo, Regent	Chocolate, forest fruits, paprika	Dry
1-A	Vineyard "Zamojska Wiśnia"	Cherries	Cherries, pit notes	Semi-sweet
2-B	Vineyard "Zamojska Gruszka"	Pears	Pear, honey, apricot	Semi-sweet
3-B	Ice wine Vineyard Zamojska	Apples	Candied orange peel, raisins, spices, baked apple	Sweet
4-A	Cuvee Rouge Vineyard Srebne	blend of Regent, Rondo, Acolon, Cabernet Cortis	Raspberries, cherries, strawberries, plums, blackberries, bilberries, smoky notes	Dry
5-A	Cuvee Rouge Vineyard Srebrna Góra	Regent, Rondo, Acolon	Rasperries, strawberries, cherries, blackberries, bilberries, cherry with pit, dried plums, smoke, forest	Dry
6-A	Vineyard Zamojska	Black Currant	Black currant, blackberries	Sweet
7-B	Cuvee Blanc Vineyard Srebrna Góra	Seyval Blanc, Johanniter, Hibernal, Solaris	Citrus, peaches, apricots, gooseberry, white currant, apples, pears, greengages, tangerines, bananas, pineapples, mango, passion fruits, flower notes, muscat, cassis, pepper	Semi-dry
8-B	Riesling Vineyard Srebrna Góra	Riesling	Apples, pears, white plums, citrus, herbal notes, spicy notes, hay, wax, butter notes, toasted notes	Dry
9-B	Chardonnay Vineyard Srebrna Góra	Chardonnay, Auxerrois	Flowers, bread, fresh apples, pears, wax, musk, vanilla, oak	Dry

10-B	Cuvee Blanc. Vineyard Srebne	Johanniter, Hibernal, Gewurztraminer, Solaris	White peaches, apricots, apples, pears, white roses, field flowers, pineapples, mango, lychees, gooseberry, tangerines	Dry
11-B	Cuvee Blanc Vineyard Półtorak	Seyval Blanc, Johanniter, Solaris, Souvignier Gris, Jutrzenka	Citrus, flowers, honey	Dry
12-B	Equus "Piaf" Cuvee Blanc	Chardonnay, Solaris, Riesling	Peaches, melons, pears, pineapples, apple jam, honey, acacia flowers	Semi-dry
13-A	Cuvee Rouge. Vineyard Półtorak	Rondo, Regent, Leon Millot, Salomea	Red fruits, spicy notes, black pepper	Dry
14-A	Equus " Vineyard Adorator"	Zweigelt, Rondo, Regent, Cabernet Cortis	Black currant, blackberries, cherries, paprika, smoked plums, tobacco	Dry
15-A	Cabernet Cortis Vineyard Niemczańska	Cabernet Cortis	Black lilac, pepper, smoked plums	Dry
16-B	Chardonnay Vineyard Niemczańska	Chardonnay	Pineapples, green apples, butter	Dry
17-B	Pinot Gris Vineyard Niemczańska	Pinot Gris	Apricots, honey, bee wax	Semi-sweet
18-A	Pinot Noir Vineyard Niemczańska	Pinot Noir	Cherries, jam, oak	Dry
19-B	Riesling Vineyard Niemczańska	Riesling	Green apples, citrus, quinces	Dry
20-B	Vincent Laura Vineyard Saint	Gewürztraminer	Roses, apricots	Semi-dry
21-B	Solaris-Riesling Vineyard Niemczańska	Solaris, Riesling	Mango, peaches, pineapples, pears, citrus, acidicity	Semi-sweet

* A – red wine, B – white wine.

foods

MDPI

Table S2. Calibration curve parameters, LOD and LOQ values of ICP-MS/ICP-OES analytical techniques.

Element	Mass	r	LOD (3s)	LOQ (10s)
Ag	107	0.99995	0.001	0.004
Al	27	0.99985	0.957	3.191
As	75	0.99996	0.005	0.017
B	11	0.99978	16.75	55.86
Ba	138	0.99995	0.003	0.011
Be	9	0.99977	0.0008	0.0026
Bi	209	0.99545	0.006	0.022
Cd	111	0.99668	0.0006	0.002
Co	59	0.99952	0.0009	0.003
Cr	52	0.99999	0.045	0.151
Cu	63	0.99923	0.198	0.660
Fe	56	0.99929	0.041	0.135
Hg	202	0.99058	0.0001	0.0004
Li	7	0.99978	0.0357	0.119
Mn	55	0.99896	0.002	0.007
Mo	95	0.99948	0.0008	0.003
Mo	98	0.99947	0.0008	0.003
Ni	60	0.99958	0.008	0.029
Pb	208	0.99999	0.002	0.008
Sb	121	0.99941	0.002	0.007
Se	78	0.99999	0.009	0.031
Sr	88	0.99889	0.006	0.021
Ti	49	0.99881	0.027	0.089
V	51	0.99987	0.002	0.007
Zn	66	0.99965	0.033	0.111
Zr	90	0.99964	0.003	0.010
**Element**	**Wavelength**	**r**	**LOD (3s)**	**LOQ (10s)**
Ca	396.8 nm	0.99992	0.234	0.780
Mg	383.2 nm	0.99963	3.870	12.90
K	404.7 nm	0.99918	73.47	244.9
Na	589.6 nm	0.99909	0.286	0.952

LOD and LOQ are in $\mu g \cdot l^{-1}$.

foods

MDPI

Table S3. Summary of data for all investigated elements according to the type of wine.

		Red wine (N = 22)			White wine (N = 31)		
**Element**	**Unit**	**Range**	**Mean**	**Median**	**Range**	**Mean**	**Median**
Al	µg·l-1	114.0 – 3430	411.9	208.0	52.00 – 3150	821.7	524.0
As	µg·l-1	0.435 – 5.410	2.168	1.640	0.542 – 13.40	4.284	3.620
B	µg·l-1	1870 – 9080	3855	2580	1400 – 8550	3866	2570
Ba	µg·l-1	52.70 – 348.0	163.3	140.0	31.00 – 318.0	127.5	102.0
Be	µg·l-1	0.0088 – 0.168	0.088	0.088	0.054 – 5.190	2.043	0.683
Cd	µg·l-1	0.085 – 6.310	0.933	0.579	0.150 – 2.070	0.727	0.657
Co	µg·l-1	0.685 – 8.170	2.351	1.775	0.957 – 11.70	3.616	3.180
Cr	µg·l-1	3.310 – 74.20	15.71	11.70	2.480 – 53.20	13.94	10.80
Cu	µg·l-1	1.980 – 169.0	48.02	29.30	10.00 – 585.0	110.4	60.10
Fe	µg·l-1	449.0 – 5170	1329	921.0	399.0 – 4870	1784	1260
Hg	µg·l-1	0.039 – 0.140	0.069	0.062	0.035 – 0.134	0.075	0.064
Li	µg·l-1	0.357 – 10.70	1.961	0.969	0.939 – 7.300	3.239	3.070
Mn	µg·l-1	774.0 – 3550	1832	1685	522.0 – 2940	1433	1430
Ni	µg·l-1	17.20 – 120.0	41.54	38.25	12.90 – 296.0	51.62	30.20
Pb	µg·l-1	3.330 – 16.60	9.443	8.945	3.310 – 30.00	14.17	12.90
Sb	µg·l-1	0.155 – 2.440	0.459	0.311	0.223 – 3.000	0.737	0.625
Se	µg·l-1	0.007 – 1.630	0.448	0.392	0.039 – 1.080	0.407	0.415
Sr	µg·l-1	187.0 – 983.0	495.4	479.5	107.0 – 720.0	362.3	317.0
Ti	µg·l-1	4.090 – 198.0	76.22	83.00	2.790 – 416.0	119.5	112.0
V	µg·l-1	0.160 – 76.20	11.19	4.170	0.234 – 166.0	25.98	8.360
Zn	µg·l-1	123.0 – 1930	606.8	512.5	74.80 – 2000	674.7	666.0
Zr	µg·l-1	1.030 – 308.0	39.75	9.805	0.674 – 354.0	68.57	39.25
Ca	mg·l-1	3.380 – 144.0	58.19	66.70	2.210 – 273.0	76.50	73.90
K	mg·l-1	1010 – 2320	1521	1440	120.0 – 3310	1160	1140
Mg	mg·l-1	46.60 – 126.0	81.23	80.00	43.00 – 450.0	86.40	69.50
Na	mg·l-1	8.320 – 122.0	33.12	12.60	10.10 – 151.0	39.71	22.40

Table S4. Minimum and maximum concentrations of elements, mean and median of determined concentrations, analyzed in wine samples depending on their origin – voivodeship.

**Voivodeship**		**Lower Silesian**	**Subcarpathian**	**Silesian**	**Lubusz**	**Lesser Poland**	**Świętokrzyskie (Holy Cross)**	**West Pomeranian**	**Lublin**
**Number of samples**		**16**	**3**	**3**	**5**	**14**	**5**	**3**	**4**
**Element**	**Unit**	**Concentration<br>Minimum – maximum<br>Mean; median**							
Al	μg·l^{-1}	131.0 – 1490<br>556.7; 337.5	132.0 – 405.0<br>300.7; 365.0	52.00 – 125.0<br>97.00; 114.0	215.0 – 595.0<br>346.8; 255.0	136.0 – 2640<br>858.4; 601.5	201.0 – 487.0<br>395.8; 412.0	158.0 – 362.0<br>234.0; 182.0	511.0 – 3430<br>2001; 2031
As	μg·l^{-1}	1.370 – 13.40<br>4.156; 3.705	0.589 – 4.710<br>2.523; 2.270	0.542 – 0.653<br>0.583; 0.553	0.828 – 4.260<br>2.273; 2.240	0.435 – 12.00<br>4.027; 3.715	1.910 – 2.810<br>2.362; 2.340	1.100 – 2.660<br>1.720; 1.400	2.630 – 9.470<br>4.990; 3.930
B	μg·l^{-1}	1920 – 8020<br>4196; 3130	1400 – 8290<br>5293; 6190	1670 – 2420<br>2057; 2080	2520 – 9080<br>5348; 5500	1990 – 6500<br>3896; 3195	1900 – 2730<br>2304; 2260	2270 – 2640<br>2403; 2300	1560 – 8550<br>3870; 2685
Ba	μg·l^{-1}	43.20 – 348.0<br>161.3; 124.5	56.10 – 109.0<br>86.43; 94.20	31.00 – 253.0<br>127.3; 97.80	45.40 – 228.0<br>114.0; 105.0	66.40 – 238.0<br>145.3; 137.0	52.70 – 102.0<br>78.76; 80.60	130.0 – 207.0<br>178.7; 199.0	86.90 – 318.0<br>197.3; 192.1
Be	μg·l^{-1}	0.054 – 4.590<br>1.932; 0.310	0.515	NA	NA	3.260 – 5.190<br>4.343; 4.580	0.183 – 0.683<br>0.487; 0.542	0.009 – 1.570<br>0.789; 0.789	NA
Cd	μg·l^{-1}	0.150 – 0.777<br>0.463; 0.472	0.241 – 0.703<br>0.412; 0.291	1.250 – 2.070<br>1.600; 1.480	0.085 – 0.728<br>0.279; 0.196	0.156 – 6.310<br>1.333; 0.813	0.178 – 1.630<br>0.941; 0.960	0.110 – 0.329<br>0.196; 0.148	0.603 – 1.160<br>0.829; 0.776
Co	μg·l^{-1}	0.779 – 4.900<br>2.661; 2.545	0.957 – 3.890<br>2.242; 1.880	1.870 – 2.600<br>2.207; 2.150	1.330 – 2.330<br>1.888; 1.900	0.685 – 11.70<br>4.274; 2.990	0.990 – 4.130<br>3.238; 3.590	1.660 – 2.560<br>1.963; 1.670	2.870 – 5.400<br>4.135; 4.135
Cr	μg·l^{-1}	7.130 – 53.20<br>16.18; 12.15	8.770 – 17.50<br>14.09; 16.00	2.480 – 16.30<br>9.727; 10.40	6.570 – 13.10<br>9.988; 10.00	3.310 – 16.70<br>10.78; 10.10	5.330 – 13.70<br>9.680; 8.800	15.20 – 20.50<br>17.07; 15.50	9.950 – 74.20<br>36.79; 31.50
Cu	μg·l^{-1}	1.980 – 169.0<br>43.82; 25.95	94.10 – 169.0<br>131.5; 131.5	15.00 – 28.00<br>21.00; 20.00	16.00 – 555.0<br>142.8; 57.00	8.000 – 585.0<br>98.65; 40.00	42.00 – 126.0<br>90.20; 98.00	44.00 – 342.0<br>148.7; 60.00	60.10
Fe	μg·l^{-1}	399.0 – 1370<br>907.1; 852.0	729.0 – 905.0<br>843.0; 895.0	493.0 – 3640<br>1687; 927.0	1080 – 2040<br>1566; 1740	449.0 – 2390<br>1460; 1410	2230 – 4410<br>3450; 3650	464.0 – 915.0<br>752.0; 877.0	1960 – 5170<br>3670; 3775
Hg	μg·l^{-1}	0.041 – 0.056<br>0.050; 0.051	0.066 – 0.090<br>0.078; 0.078	NA	0.045 – 0.066<br>0.058; 0.064	0.035 – 0.124<br>0.078; 0.085	NA	NA	0.057 – 0.140<br>0.107; 0.115
Li	μg·l^{-1}	0.918 – 10.70<br>4.123; 3.620	1.280	0.939 – 3.250<br>2.073; 2.030	0.879 – 2.580<br>1.730; 1.730	0.357 – 4.260<br>1.546; 0.947	0.832 – 4.600<br>3.000; 3.110	1.020 – 3.820<br>2.070; 1.370	NA
Mn	μg·l^{-1}	700.0 – 3550<br>1743; 1560	1090 – 2110<br>1723; 1970	670.0 – 1640<br>1050; 841.0	603.0 – 2280<br>1258; 1030	999.0 – 2940<br>1843; 1915	1060 – 1660<br>1336; 1390	1430 – 1790<br>1597; 1570	522.0 – 2160<br>1241; 1142

Ni	μg·l⁻¹	25.60 – 296.0<br>71.46; 49.30	52.20 – 78.10<br>62.27; 56.50	12.90 – 120.0<br>54.10; 29.40	21.50 – 45.40<br>29.90; 25.40	17.20 – 63.60<br>30.91; 25.15	23.10 – 29.60<br>26.84; 26.90	33.70 – 41.90<br>37.10; 35.70	30.20 – 85.10<br>48.50; 39.35
Pb	μg·l⁻¹	3.310 – 19.80<br>12.30; 11.30	6.340 – 10.90<br>7.973; 6.680	7.280 – 16.40<br>11.79; 11.70	4.380 – 14.40<br>11.09; 12.90	3.330 – 16.50<br>10.75; 10.41	14.00 – 26.70<br>21.56; 21.60	7.900 – 9.340<br>8.690; 8.830	6.800 – 30.00<br>12.82; 7.240
Sb	μg·l⁻¹	0.217 – 1.490<br>0.590; 0.476	0.440 – 0.633<br>0.527; 0.509	0.223 – 0.381<br>0.305; 0.310	0.155 – 0.511<br>0.375; 0.388	0.226 – 0.951<br>0.553; 0.601	0.195 – 0.558<br>0.392; 0.381	0.282 – 0.743<br>0.449; 0.321	1.210 – 3.000<br>2.023; 1.940
Se	μg·l⁻¹	0.097 – 0.508<br>0.325; 0.367	0.427 – 0.491<br>0.459; 0.459	0.114	0.472 – 1.630<br>1.061; 1.080	0.018 – 1.260<br>0.476; 0.532	0.131 – 0.369<br>0.214; 0.178	0.007 – 0.084<br>0.046; 0.046	0.450 – 0.786<br>0.588 ; 0.557
Sr	μg·l⁻¹	164.0 – 983.0<br>466.8; 444.0	314.0 – 483.0<br>371.3; 317.0	245.0 – 684.0<br>431.0; 364.0	107.0 – 596.0<br>397.2; 385.0	216.0 – 720.0<br>425.0; 426.5	187.0 – 465.0<br>288.2; 260.0	389.0 – 508.0<br>442.0; 429.0	182.0 – 681.0<br>388.0; 344.5
Ti	μg·l⁻¹	2.790 – 280.0<br>113.3; 93.50	4.740 – 100.0<br>37.13; 6.640	112.0 – 136.0<br>124.0; 124.0	4.750 – 173.0<br>60.21; 6.310	4.990 – 416.0<br>99.52; 82.50	106.0 – 234.0<br>190.0; 203.0	86.00 – 102.0<br>91.67; 87.00	18.30 – 77.70<br>41.65; 35.30
V	μg·l⁻¹	2.910 – 166.0<br>27.56; 8.010	1.690 – 8.360<br>4.740; 4.170	0.232 – 1.170<br>0.545; 0.234	0.160 – 10.90<br>4.428; 4.550	0.260 – 36.70<br>14.83; 13.60	1.150 – 7.470<br>3.618; 2.890	1.240 – 4.290<br>2.660; 2.450	36.60 – 163.0<br>85.62; 71.45
Zn	μg·l⁻¹	370.0 – 969.0<br>678.6; 669.0	221.0 – 584.0<br>450.3; 546.0	390.0 – 2000<br>948.3; 455.0	373.0 – 814.0<br>511.2; 466.0	123.0 – 1930<br>724.1; 666.0	300.00 – 1000<br>627.0; 699.0	384.0 – 1080<br>740.3; 757.0	74.80 – 479.0<br>290.4; 304.0
Zr	μg·l⁻¹	0.674 – 45.80<br>17.39; 10.90	12.60 – 36.20<br>21.20; 14.80	NA	10.10 – 19.20<br>13.83; 12.20	3.220 – 354.0<br>97.71; 46.50	1.030 – 67.00<br>41.79; 41.30	1.290 – 3.900<br>2.493; 2.290	118.0 – 308.0<br>208.0; 203.0
Ca	mg·l⁻¹	2.290 – 170.0<br>67.68; 65.05	2.810 – 73.90<br>26.82; 3.750	86.50 – 102.0<br>94.60; 95.30	2.480 – 129.0<br>44.27; 3.380	3.490 – 273.0<br>70.55; 65.50	78.00 – 163.0<br>133.8; 141.0	64.70 – 83.30<br>71.80; 67.40	2.210 – 57.00<br>27.79; 25.98
K	mg·l⁻¹	120.0 – 1880<br>1154; 1225	664.0 – 1300<br>975.7; 963.0	998.0 – 1330<br>1123; 1040	768.0 – 1450<br>1098; 1030	927.0 – 2280<br>1437; 1315	1010 – 1740<br>1356; 1420	598.0 – 2320<br>1476; 1510	1380 – 3310<br>1965; 1585
Mg	mg·l⁻¹	43.90 – 126.0<br>76.64; 79.95	44.60 – 111.0<br>81.83; 89.90	54.10 – 450.0<br>195.5; 82.30	60.60 – 128.0<br>93.92; 101.0	43.00 –117.0<br>78.95; 84.15	48.90 – 69.50<br>56.66; 52.20	59.10 – 80.90<br>67.30; 61.90	49.20 – 134.0<br>86.85; 82.10
Na	mg·l⁻¹	8.320 – 21.30<br>15.84; 16.20	22.10 – 30.40<br>26.25; 26.25	NA	10.10 – 14.80<br>11.93; 10.90	8.540 – 42.80<br>22.82; 23.15	NA	NA	67.90 – 151.0<br>115.2; 121.0

* NA – not analysed.
